# Proteinized Surface Remodeling Decouples Environmental Stability from Intracellular Degradation in Oxidation-Prone 2D Nanoborophene

**DOI:** 10.1021/acsnano.6c11186

**Published:** 2026-09-09

**Authors:** Pranay Saha, Shraddha Krishnakumar, Teresa Aditya, Nada Maher, Aysenur Yardim, Oguzhan Colak, David Skrodzki, Sung Hyun Cho, Xu Feng, Dongjun Shin, Dipanjan Pan

**Affiliations:** † Department of Nuclear Engineering, 8082 The Pennsylvania State University, University Park, Pennsylvania 16802, United States; ‡ Huck Institutes of the Life Sciences, 101 Huck Life Sciences Building, University Park, Pennsylvania 16802, United States; § Department of Biomedical Engineering, 311285 The Pennsylvania State University, University Park, Pennsylvania 16802, United States; ∥ Department of Materials Science and Engineering, 311285 The Pennsylvania State University, University Park, Pennsylvania 16802, United States; ⊥ Department of Chemistry, The Pennsylvania State University, University Park, Pennsylvania 16802, United States; # Materials Research Institute, The Pennsylvania State University, University Park, Pennsylvania 16802, United States; ∇ Center for Infectious Disease Dynamics, The Pennsylvania State University, University Park, Pennsylvania 16802, United States; ○ Department of Bioengineering, Institute of Natural Sciences, Ege University, Izmir 35100, Türkiye; ◆ Surface Analysis Facility, 137753 University of Delaware, Newark, Delaware 19716, United States; ¶ Tomocube Inc., San Diego, California 92108, United States

**Keywords:** 2D nanoborophene, protein-assisted exfoliation, borophene, proteinized borophene, surface modification, degradation kinetics, biointerfacial sheath

## Abstract

Borophene (BPH), a monolayer boron allotrope, exhibits outstanding electrical and mechanical properties but remains intrinsically constrained by its excessive susceptibility to oxidative degradation in aqueous and physiological environments. This study demonstrates that protein-assisted exfoliation using human serum albumin (HSA) establishes a dynamic biointerfacial sheath (BIS) that decouples environmental stability from intracellular degradation. HSA forms a robust BIS via multivalent hydrophobic, electrostatic, and thiol-mediated interactions, driving exfoliation into ultrathin nanosheets while suppressing air- and serum-induced oxidation. Proteinized BPH (PBPH) shows enhanced extracellular colloidal and chemical stability; however, upon cellular uptake, the same interface promotes rapid reactive oxygen species-driven degradation, leading to lattice fragmentation, loss of crystallinity, and conversion into soluble boronate species. Multimodal analyses confirm accelerated degradation kinetics and environmental stability of PBPH. These results highlight the protein corona as an active regulator of nanomaterial fate, enabling programmable decoupling of extracellular persistence from intracellular clearance in oxidation-prone two-dimensional systems.

## Graphical abstract

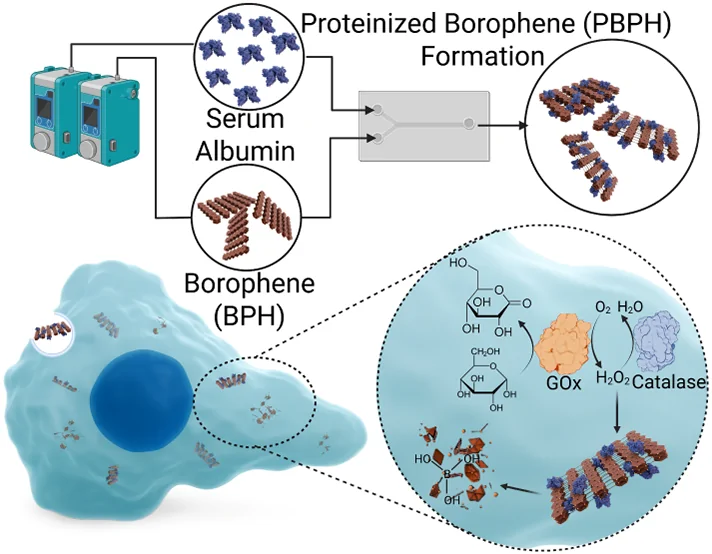

## Introduction

Two-dimensional (2D) nanomaterials have become integral to biomedical technologies owing to their tunable surface chemistry, ultrathin dimensions, and distinctive physicochemical properties. Materials such as graphene, black phosphorus, and transition metal dichalcogenides (TMDs) have enabled advances in biosensing, drug delivery, theranostics, and tissue engineering through high surface area, electronic tunability, and dynamic reactivity.^1–11^ Within this family, borophene (BPH), an atomically thin boron allotrope, has emerged as a distinctive metallic conductor characterized by low atomic mass, in-plane mechanical anisotropy, and Dirac-like electronic behavior.^12–20^ The β_12_ and χ_3_ polymorphs exhibit high carrier mobility, negative Poisson’s ratio, and mechanical strength surpassing that of graphene along specific ridgelines, supporting applications in flexible electronics, electrochemical sensing, and adaptive nanomechanical systems.^21–23^

Despite these advantages, BPH exhibits pronounced susceptibility to oxidative degradation in aqueous and physiological environments, similar to oxidation-prone two-dimensional materials (e.g., phosphorene).^20,24–26^ Its electron-rich surface and high reactivity drive rapid formation of boron oxides (e.g., B_2_O_3_) and organoboronic acids, compromising structural integrity, biocompatibility, and functional lifespan. Conventional surface passivation approaches, including PEGylation and polymer grafting, are ineffective or detrimental owing to BPH’s inherent instability. Therefore, effective stabilization requires controlling BPH transformation within biological environments rather than solely inhibiting oxidation.

In biological fluids, nanomaterials acquire dynamic protein or biomolecular coronas that govern colloidal behavior, cellular recognition, and chemical persistence.^11,27–32^ For oxidation-prone 2D materials, these biointerfaces critically influence stability and degradation.^10,33–36^ Protein-assisted exfoliation (PAE) offers a biologically compatible stabilization. Unlike graphene, BPH is intrinsically electron-deficient owing to boron’s trivalent configuration (2s^2^2p^1^), enabling multicenter bonding and contributes to high conductivity and chemical reactivity. Human serum albumin (HSA), an abundant amphiphilic and redox-active protein enriched in cysteine residues (∼7%), strongly interacts with reactive nanosurfaces via hydrophobic, electrostatic, and sulfur-mediated interactions.^37–40^ Therefore, we hypothesized that HSA stabilizes and actively remodels BPH via proteinized surface exfoliation, forming a dynamic biointerfacial sheath (BIS) (Figure 1a) that regulates BPH transformation and trafficking in biological systems.

**Figure 1:**
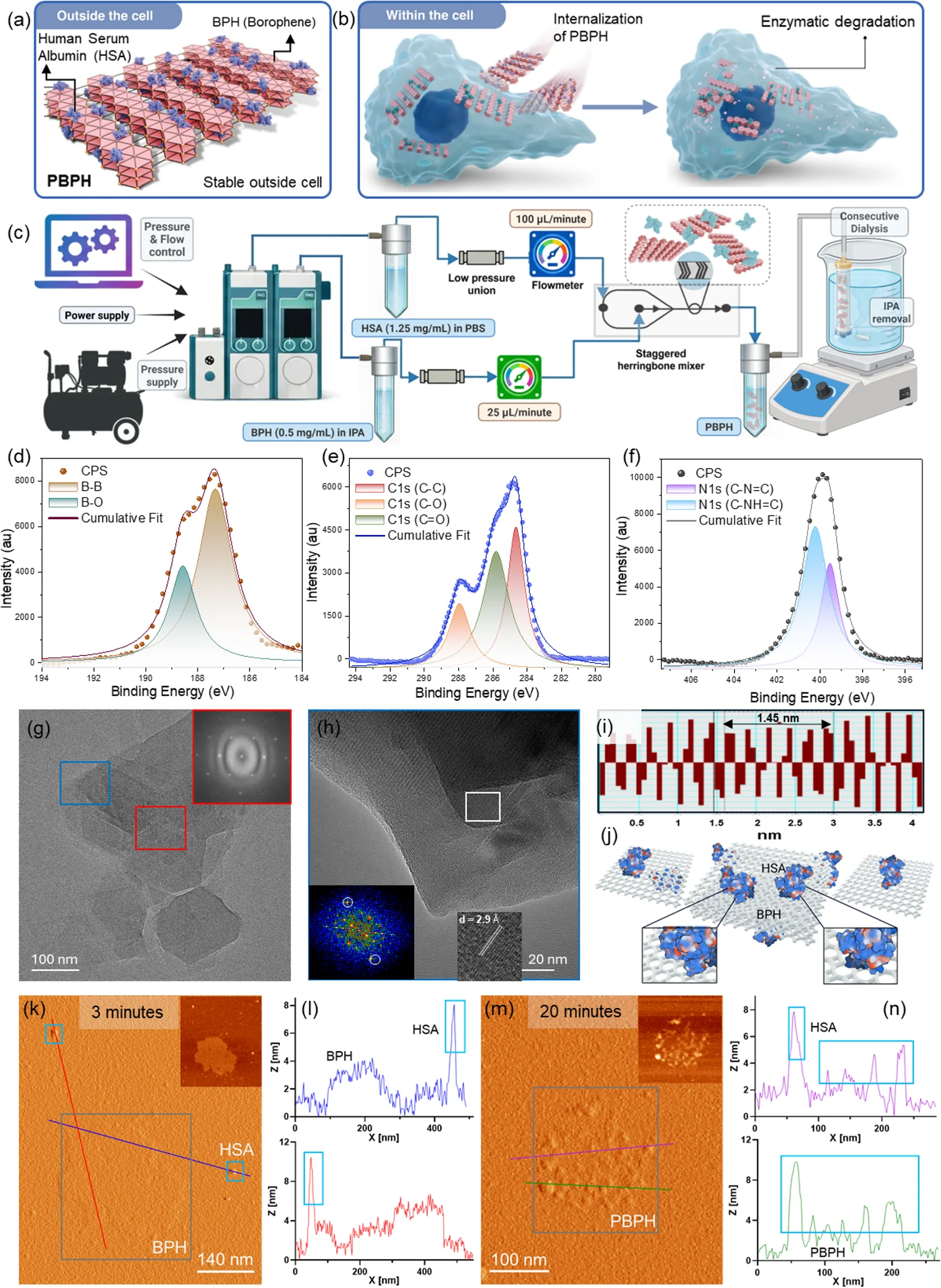
(a) Schematic of PBPH showing HSA-induced protein-assisted exfoliation (PAE) of borophene (BPH) and formation of a biointerfacial sheath (BIS) that enhances extracellular stability. (b) Intracellular degradation pathway of PBPH, where enzymatic ROS generation accelerates oxidative fragmentation relative to pristine BPH. (c) Microfluidic synthesis of PBPH. (d–f) High-resolution XPS spectra of PBPH with peak deconvolution of (d) B 1s, (e) C 1s, and (f) N 1s, confirming successful protein functionalization. (g, h) TEM images of PBPH showing preserved lattice fringes (∼2.9 Å); inset in (g) shows the diffraction pattern from the marked region, and (h) shows a magnified view with corresponding FFT confirming retained crystallinity. (i) Quantitative *d*-spacing analysis from inverse FFT. (j) Structural model of HSA adsorption on BPH. (k–n) AFM topographic and phase images with corresponding line profiles, revealing protein adsorption, surface morphology, and protein-induced structural modulation during PBPH formation.

This study evaluates the hypothesis that protein-assisted exfoliation represents a biologically compatible strategy to stabilize and functionally regulate borophene in physiological environments. Specifically, we examine the interactions between HSA and borophene leading to the formation of proteinized borophene (PBPH) as a dynamic BIS that modulates the material’s physicochemical properties, interfacial reactivity, and oxidative stability. We further investigate whether this protein-mediated reconstitution enables a context-dependent behavior, stabilizing borophene under extracellular conditions while permitting controlled degradation following cellular internalization. By framing protein adsorption as an active regulator rather than a passive coating, this study establishes proteinized surface exfoliation as a biologically informed strategy to engineer reactive 2D materials with coupled stability, transformation, and biological clearance.

The biological identity of nanoparticles is strongly influenced by the formation of protein coronas, which regulate stability, cellular uptake, and biological fate.^41–44^ However, these studies have primarily focused on nanomaterials that are intrinsically stable under physiological conditions. In contrast, borophene is a transient two-dimensional material that undergoes rapid oxidative degradation in biological environments, making interfacial engineering essential rather than optional. The engineered HSA biointerfacial sheath (BIS) therefore serves not only to modify biological interactions but also to preserve borophene during extracellular transport while enabling controlled intracellular degradation. This environmentally responsive regulation distinguishes the present strategy from conventional protein-corona engineering and establishes the biointerface as an active regulator of borophene fate.

HSA-mediated stabilization provides a strategy for transient, biodegradable nanomaterials that combine controlled extracellular function with programmed intracellular ROS-triggered disassembly, where proteinization protects the BPH surface while facilitating cell entry (Figure 1b). This self-limiting behavior reduces bioaccumulation and aligns with emerging nanoregulation frameworks emphasizing safety and degradability. Overall, this study bridges boron nanochemistry with cellular redox biology, enabling development of borophene-based bionanodevices that remain transient, reactive, and responsive to the oxidative biological systems.

## Results

### Rationale-Driven Formation of HSA-Stabilized Proteinized Borophene (PBPH) Biointerfacial Sheath (BIS) Complexes

Initial assessment of borophene degradation employed UV–vis, Raman and FTIR spectroscopy. UV–vis spectroscopy was used to monitor the oxidative degradation of borophene under varying H_2_O_2_ concentrations. At higher oxidant levels, BPH exhibited a rapid and pronounced decrease in absorbance over time, accompanied by increased dispersion transparency, indicating accelerated degradation and conversion to soluble borate species (Figure S1a). Conversely, lower H_2_O_2_ concentrations yielded gradual attenuation of absorbance (Figure S1b), reflecting slower oxidation kinetics and delayed structural breakdown. These results confirm a concentration-dependent degradation pathway, wherein increased ROS availability directly enhances borophene oxidation and dissolution. Raman spectra of pristine BPH showed characteristic vibrational modes that progressively attenuated upon H__2__O__2__ exposure, indicating lattice disruption and loss of structural integrity (Figure S2a). Consistently, FTIR revealed the emergence of strong B–O stretching bands (∼1350–1450 cm^–1^) and broad O–H absorptions (∼3200–3500 cm^–1^), confirming oxidation to boronic/borate species and formation of hydrated oxide products (Figure S2b). To mitigate the intrinsic oxidative instability of BPH, multiple biomolecules including low-molecular-weight amino acids such as cysteine,^10^ were evaluated under aqueous and ROS-rich conditions (Figure S3a,b). Comparative analysis revealed rapid degradation and turbidity loss in the presence of low-molecular-weight amino acids, indicating limited surface protection. By contrast, HSA-treated systems retained dispersion stability (Figure S3b) and structural integrity over extended durations (Figure S3c), demonstrating superior resistance to oxidation. This enhanced stability is attributed to the macromolecular architecture of HSA, enabling multivalent interactions, surface adaptability, and formation of a dynamic BIS that effectively limits ROS accessibility to the borophene surface. These findings establish the rationale for engineering a protein-based BIS to modulate borophene stability and reactivity.

Accordingly, BPH nanosheets were synthesized via sonication-assisted liquid-phase exfoliation of crystalline boron and dispersed in IPA (0.5 mg mL^–1^). The BPH suspension and HSA solution (1.25 mg mL^–1^ in PBS) were coinjected into a pressure-driven microfluidic staggered herringbone mixer (25 and 100 μL min^–1^, respectively), enabling rapid and homogeneous interfacial mixing (Figure 1c). This process induced protein-assisted exfoliation (PAE) and formation of a BIS via van der Waals, electrostatic, and hydrophobic interactions,^45–48^ consistent with XPS and AFM analyses (Figure 1d–n). Dialysis removed IPA, yielding a stable aqueous PBPH dispersion without surfactants or oxidation-prone functionalization. Nanostructural evolution of BPH and PBPH was confirmed by HRTEM, cryo-TEM, and AFM.

### Protein-Assisted Exfoliation Reconfigures BPH into Biointerfacial Sheath-Stabilized Nanosheets

X-ray photoelectron spectroscopy (XPS), high-resolution transmission electron microscopy (HRTEM), atomic force microscopy (AFM), and cryogenic transmission electron microscopy (cryo-TEM) were employed to characterize the nanostructural evolution of pristine BPH and PBPH biointerfacial sheath complex. Solid-state ^11^B NMR of PBPH exhibited a characteristic resonance at ∼12.3 ppm, confirming the presence of boron in a predominantly B–O coordinated environment consistent with surface-modified borophene (Figure S4). XPS of PBPH retains the characteristic B–B and B–O peaks of BPH, confirming preservation of its elemental framework (Figure 1d), while the emergence of C 1s and N 1s signals (e.g., C–C, C–N and amide functionalities) verifies successful HSA adsorption on the surface (Figure 1e,f). HRTEM and cryo-TEM of pristine BPH in vitrified ice revealed multilayered stacks with limited lateral separation, confirming its aggregation tendency in aqueous media (Figure S5a,b). Conversely, PBPH samples exhibited distinctly thinner nanosheets (Figure 1g) with interplanar lattice spacings of ∼0.29 nm and diffraction patterns consistent with β_12_ polymorphs, confirming characteristic crystallinity (Figure 1h,i). SAXS profiles displayed a broad peak at q ≈ 1.9 Å^–1^, corresponding to an average interatomic spacing of d ∼2.9 Å, consistent with HRTEM measurements for both BPH and PBPH, indicating preservation of short-range structural correlations after protein adsorption (Figure S6). Upon addition of H__2__O__2__, PBPH exhibits progressive attenuation of this feature, reflecting oxidative degradation and loss of local structural coherence. Schematic representation (Figure 1j) of HSA surface adsorption was obtained via AFM topography at 3 min time-point reaction between BPH and HSA, revealing BPH nanosheets with low surface roughness and nanoscale thickness (Figure 1k), where HSA is distinguishable from BPH via height profiles (Figure 1l). PBPH height profiles at 20 min time-point exhibited an increase in overall surface roughness and thickness from ∼2–4 nm (BPH) to ∼5–10 nm (PBPH), consistent with protein-layer deposition on BPH (Figure 1m–n).

### Biointerfacial Sheath-Driven Exfoliation and Structural Reorganization of Borophene across Scales

Following HSA interaction, the partially amorphous and low-electron-dense BIS obscured the fine lattice features of PBPH, particularly at sheet edges and thinner regions, with lattice visibility restricted to thicker inner regions (Figure 1g,h). To overcome these limitations and preserve hydrated protein structure, time-resolved cryo-TEM was employed, with sample vitrification illustrated in Figure 2a. Cryo-TEM demonstrated that HSA interaction induced protein-assisted exfoliation (PAE) of BPH, with initial multilayer stacks at 5 min transitioning to partially delaminated sheets at 10 min and pronounced edge separation by 15 min. At 30 min, well-exfoliated PBPH nanosheets enveloped by halo-like BIS with distinct “soft” and “hard” domains were observed, indicating sustained protein-mediated stabilization and progressive structural reorganization into discrete layers (Figure 2b–e). Probable interaction modalities are illustrated in Figure 2f.

**Figure 2:**
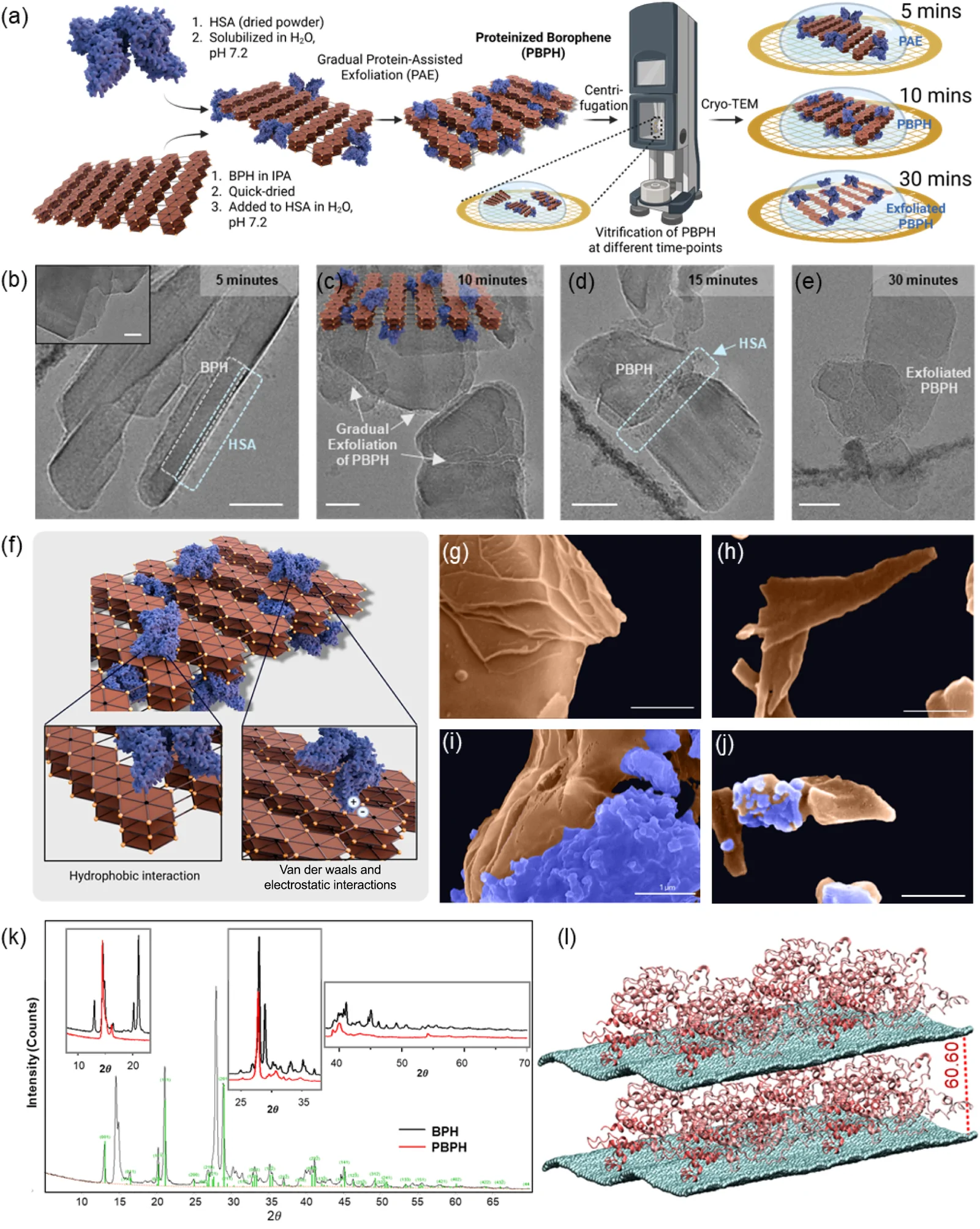
(a) Schematic of cryo-TEM sample preparation showing HSA-mediated protein-assisted exfoliation (PAE) of BPH and vitrification prior to imaging. (b–e) Time-resolved cryo-TEM images acquired at 5, 10, 15, and 30 min, revealing progressive exfoliation and delamination of BPH upon HSA interaction. (f) Schematic of the intermolecular interactions driving PBPH formation, including hydrophobic, van der Waals, and electrostatic forces. (g, h) SEM images of pristine BPH before and after extended probe sonication. (i, j) SEM images of PBPH before and after extended probe sonication, showing enhanced exfoliation and reduced stacking in the presence of HSA. BPH and HSA are pseudocolored brown and blue, respectively. (k) XRD patterns of BPH and PBPH, indicating protein-induced disruption of interlayer stacking, partial exfoliation, reduced crystallite size, and increased amorphization. (l) Atomistic molecular dynamics model of HSA adsorbed between parallel BPH sheets, showing formation of a stabilizing protein adlayer with an interlayer spacing of ∼60.6 Å.

Scanning electron microscopy (SEM) corroborated this transition from stacked BPH layers to thinner, delaminated sheets in the presence of HSA. Pristine BPH samples exhibited crumpled multilayer flakes with nano- to micrometer-scale lateral dimensions, whereas PBPH displayed uniformly dispersed, unilamellar 2D structures with globular protein bulges and visible delamination fronts. These characteristic persisted after extended probe sonication, highlighting enhanced exfoliation efficiency, reduced restacking, and strong interfacial interactions (Figure 2g–j).

X-ray diffraction (XRD) patterns of pristine BPH displayed sharp low-angle reflections (2θ ≈ 12°–15°) associated with interlayer stacking and additional midangle peaks (2θ ≈ 25°–35°), indicating preserved in-plane crystallinity and ordered lamellar packing (Figures 2k, S7). Conversely, PBPH exhibited attenuation or shifts in the low-angle stacking peak, broadening of midangle reflections, and the emergence of a broad feature around 2θ = 20°–23° attributed to amorphous protein, collectively indicating interlayer expansion, partial exfoliation, reduced crystallite coherence, and increased structural disorder upon protein functionalization (Figure 2k). All-atom molecular dynamics (MD) simulations of HSA adsorption on BPH surfaces (Figure 2l) constructed from a validated crystal model reproduced interfacial orientation, surface coverage, and protein-mediated interlayer spacing (∼6 nm) consistent with experimental observations (AFM, Figure 1n), indicating noncovalent interactions in BIS stabilization. The all-atom MD simulations represent a simplified model of the engineered HSA-borophene interface and were intended to provide atomistic insight into HSA adsorption, orientation, and interfacial stabilization rather than to reproduce the heterogeneous and dynamic protein corona formed under physiological conditions. In vivo, competitive adsorption and exchange of multiple plasma proteins continuously remodel the nanoparticle surface. Nevertheless, the experimentally observed BIS was independently validated by ITC, AFM, XPS, and stability studies, while the simulations provide complementary mechanistic insight into the noncovalent interactions governing HSA adsorption and stabilization of borophene

### Quantitative Characterization of the Albumin Biointerfacial Sheath

To determine whether borophene stabilization was specific to HSA rather than nonspecific protein adsorption, we compared HSA with BSA, PEG, and blood serum (IRB: STUDY00028808). To quantitatively evaluate the interaction between HSA and borophene, isothermal titration calorimetry (ITC) was performed by titrating HSA (100 μM) into a borophene suspension (0.1 mg mL^–1^). The thermograms displayed progressive saturation with increasing HSA concentration and were well fitted using an independent binding model, yielding an apparent dissociation constant (*K*_d_) of 7.65 × 10^–8^ M, an apparent binding stoichiometry (n) of 2.02, ΔH of 4.53 kcal mol^–1^, and ΔS of 47.5 cal mol^–1^ K^–1^ (Figure 3a). These results demonstrate a strong, entropy-driven interaction between HSA and borophene, providing quantitative evidence for the formation of a stable HSA biointerfacial sheath. ITC revealed a strong, saturable interaction between HSA and borophene (Figure 3a), whereas PEG produced nonsignificant heat changes consistent with minimal binding. Blood serum generated a complex, heterogeneous thermogram without a clear saturation profile, reflecting competitive adsorption among multiple serum components (Figure 3b). These results support preferential formation of a defined HSA biointerfacial sheath compared with PEG or spontaneously formed serum coronas. This interaction is likely driven by a combination of van der Waals, electrostatic, and hydrophobic interactions, together with the solvent-accessible Cys34 thiol, which can potentially form B–S bonds with borophene.^7,10,49,50^

**Figure 3:**
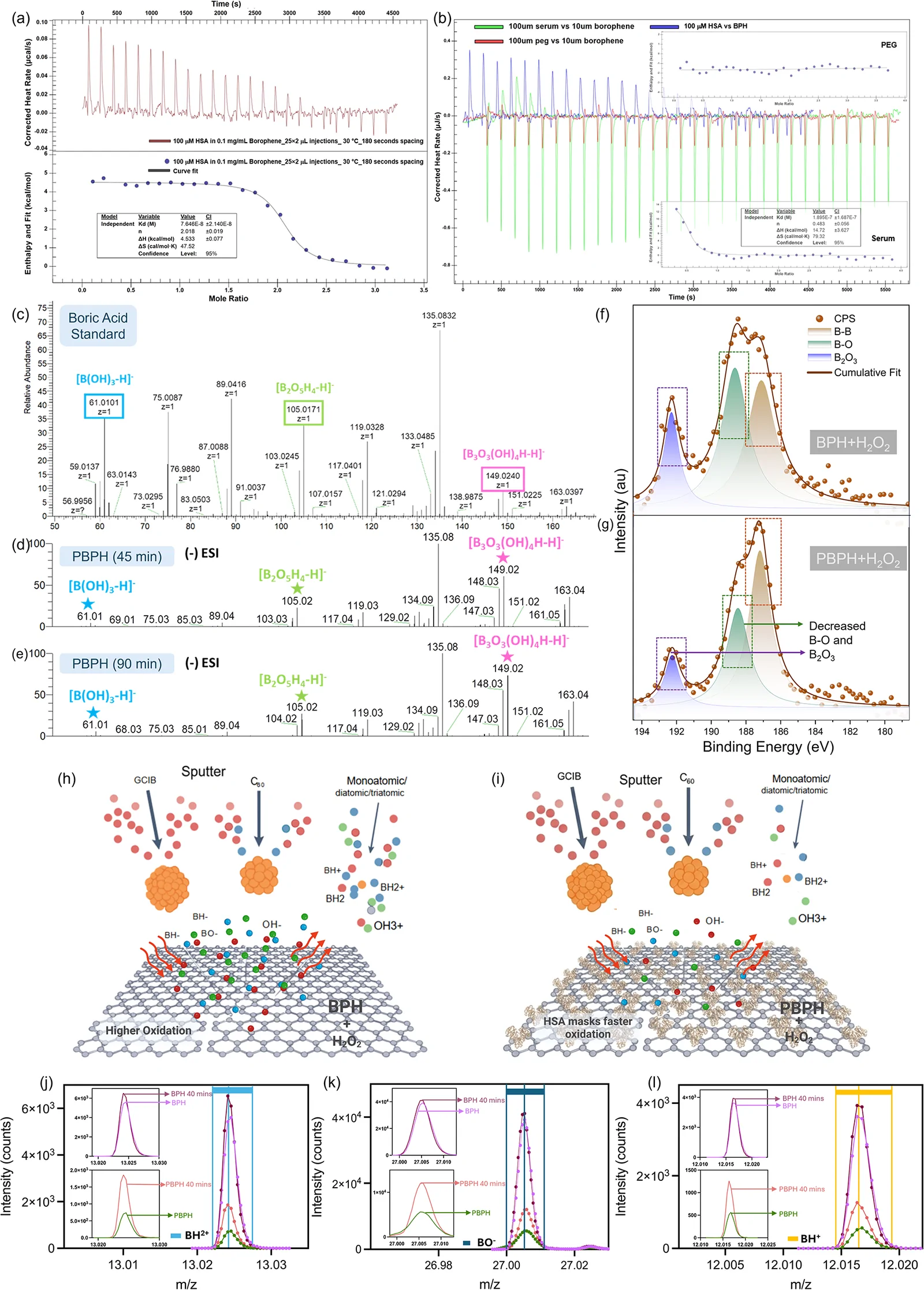
(a) Representative ITC thermogram (top) and fitted binding isotherm (bottom) for HSA (100 μM) titrated into borophene (0.1 mg mL^–1^). (b) Overlay of raw ITC thermograms for HSA, PEG, and blood serum with borophene; insets show the corresponding integrated isotherms for PEG and serum. (c–e) HPLC–MS profiles collected after 45 and 90 min incubation of PBPH, showing time-dependent evolution of boron-containing species during oxidative degradation. (f, g) High-resolution XPS B 1s spectra of (f) pristine BPH and (g) PBPH after H_2_O_2_ exposure, deconvoluted into B–B, B–O, and B_2_O_3_ components. PBPH exhibits a lower contribution from oxidized boron species, indicating moderated oxidation kinetics. (h, i) Schematic of oxidative degradation under gas cluster ion beam/TOF-SIMS conditions for (h) BPH and (i) PBPH, highlighting attenuated oxidation in the presence of the HSA biointerfacial sheath. (j–l) High-resolution TOF-SIMS spectra showing isotopic envelopes of characteristic boron fragments (BH_2_^+^, BH^+^, and BO^–^) for BPH, PBPH, and their 40 min reaction mixtures, confirming retention of boron framework fragments during controlled oxidative transformation.

Notably, HSA contains a single solvent-accessible free thiol (Cys34), which may contribute to stable borophene binding through boron–sulfur (B–S) interactions in addition to noncovalent adsorption. Engineering HSA to expose additional thiol groups through controlled disulfide reduction may provide a strategy for further strengthening the biointerface and will be investigated in future studies

### ROS-Driven Differential Degradation Kinetics in BPH and PBPH with Preserved Structural Integrity

Oxidative degradation pathways and BIS-mediated protection in PBPH were investigated employing a multimodal analytical framework combining HPLC-MS, XPS, ToF-SIMS, and a self-reporting curcumin–borate assay. HPLC–MS, calibrated against boric acid standards (Figure 3c), revealed time-dependent emergence of boron-containing degradation products in PBPH (45–90 min), confirming progressive oxidation into soluble borate species (Figure 3d,e). High-resolution XPS further demonstrated that pristine BPH is dominated by oxidized boron environments (B–O/B_2_O_3_ ∼ 192.8 eV) (Figure 3f), whereas PBPH retained a higher fraction of B–B bonding (∼187.6 eV) with attenuated oxidized components (Figure 3g), indicating that HSA association moderated surface oxidation kinetics.

ToF-SIMS provided molecular-level insights into fragmentation behavior. Pristine BPH exhibited extensive ion emission owing to direct surface exposure, generating abundant mono- and polyatomic boron and oxidized fragments (Figure 3h–i). By contrast, PBPH exhibited attenuated sputtering yields, as the HSA-derived BIS partially shielded the surface, limiting ion ejection and reducing the formation of oxidized boron fragments. Furthermore, BPH exhibited extensive oxidation with formation of BO^–^, BH^2+^, BH^+^, and other hydroxylated boron species alongside oxidant-derived ions (Figure 3j–l). By contrast, PBPH showed reduced intensities of these oxidized fragments, confirming that the BIS limits oxidant accessibility and suppresses rapid surface degradation. These findings were corroborated by isotopic signatures (BO^–^, BH^2+^, BH^+^), which persisted across conditions, indicating retention of boron framework fragments during controlled transformation.

Complementary optical validation using the curcumin–borate (rosocyanine) self-reporting assay (Figure 4a–g) confirmed differential degradation. Oxidative degradation of BPH and PBPH generated borate species that induce a characteristic yellow-to-reddish-orange transition (Figure 4a). Time-resolved absorbance and paper-strip readouts revealed a more rapid and spatially pronounced color evolution for BPH compared with PBPH, indicating greater borate formation in the pristine system (Figure 4b). Curcumin-impregnated paper strips were prepared to act as a reactive substrate for borate detection (Figure 4c). Upon exposure to degradation products, borate species generated from BPH or PBPH reacted with curcumin to form the rosocyanine complex, producing a visible color change. As expected, pristine BPH underwent faster oxidation, generated higher concentrations of borate species, and yielded rapid and intense color development (Figure 4d), whereas PBPH showed slower and weaker color evolution owing to BIS-mediated protection. The experimental paper-strip readouts in Figure 4e corroborate this trend, showing a time-dependent transition from yellow to reddish orange, with BPH exhibiting earlier onset and stronger coloration compared with the more gradual and subdued response of PBPH, consistent with attenuated degradation kinetics in the proteinized system. Quantitative image analysis showed a progressive decrease in green channel intensity from ∼157 to 78 a.u. for BPH and from ∼184 to 109 a.u. for PBPH over 60 min, confirming slower degradation in the proteinized system. Kinetic analysis (ln­(I/I_0_) vs time) followed pseudo-first-order behavior with strong linear fits (R^2^ ≈ 0.967 for BPH and R^2^ ≈ 0.9917 for PBPH), yielding apparent rate constants of k ≈ 0.0107 min^–1^ for BPH and k ≈ 0.0085 min^–1^ for PBPH (Figure 4f), thereby indicating attenuated oxidation kinetics upon HSA association. This trend was further supported by ΔE analysis, which showed a higher and more rapid increase in color contrast for BPH relative to PBPH over time (Figure 4g). Collectively, these results confirm that although both systems underwent oxidative conversion to borate species, the HSA-derived BIS significantly delayed degradation by limiting interfacial accessibility and moderating oxidative reactivity.

**Figure 4:**
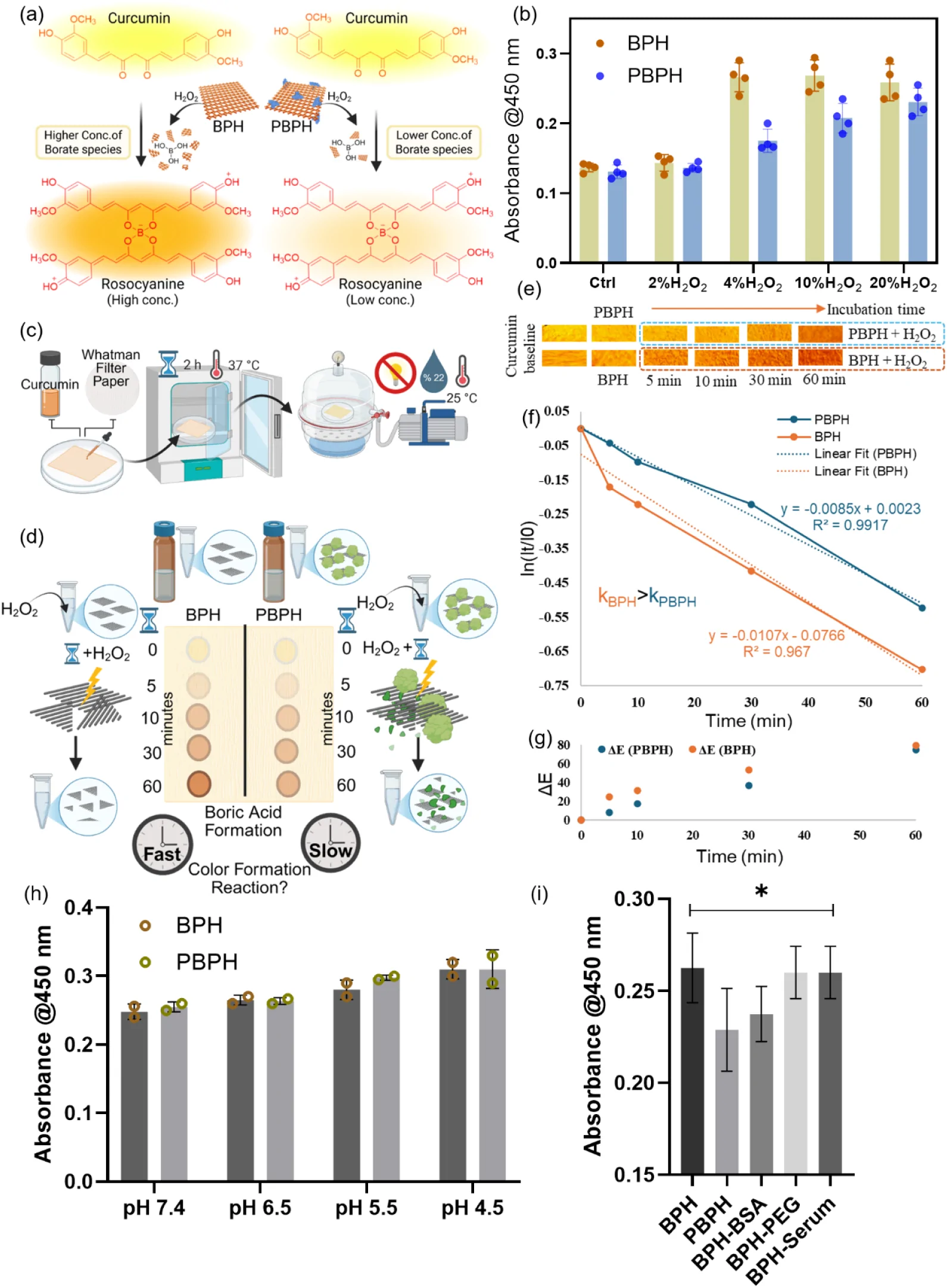
(a) Schematic of the curcumin-borate optical assay, where oxidative degradation of BPH generates borate species that react with curcumin to form the rosocyanine complex. (b) Comparative absorbance evolution of curcumin–borate complexes for BPH and PBPH, showing greater degradation of pristine BPH. (c) Preparation of curcumin-impregnated paper strips for visual degradation monitoring. (d) Representative paper-strip readouts over time, illustrating the temporal evolution of borate formation and comparative degradation behavior. (e) Time-resolved color transition from yellow to reddish-orange, providing a visual indicator of borate generation. (f) Kinetic analysis of degradation based on optical measurements, showing ln­(I/I_0_) versus time with linear fits; pristine BPH exhibits a higher degradation rate constant than PBPH. (g) Time-dependent color difference (ΔE) analysis from the paper-strip assay, highlighting distinct degradation kinetics between BPH and PBPH. (h) Rosocyanine assay showing enhanced oxidation resistance of PBPH compared with pristine BPH under physiological and acidic pH. (i) Measuring degraded boronate species with rosocyanine assay comparing.

Consistent with previous ITC results, the borate-curcumin (rosocyanine) assay demonstrated that PBPH and BPH exhibited similar stability at different pH (Figure 4h) which is statistically not significant which paves way for endosomal degradation of PBPH intracellularly. On the other hand, both HSA- and BSA-coated borophene exhibited markedly greater resistance to oxidation than pristine borophene, whereas PEG and serum provided negligible protection (Figure 4i). These findings are consistent with the dynamic nature of spontaneous protein coronas, which undergo continuous protein exchange and form heterogeneous interfaces. In contrast, the pre-engineered HSA BIS provides a defined, reproducible interface that suppresses premature extracellular oxidation while preserving controlled intracellular degradability. Collectively, these results demonstrate that albumin, rather than nonspecific protein adsorption, is the primary determinant of borophene stabilization under extracellular conditions.

### In Vitro Cellular Uptake and Degradation Dynamics of BPH and PBPH

Pharmacological inhibition and time-resolved ICP-MS/OES were employed to evaluate cellular uptake, intracellular processing, and degradation of BPH and PBPH in A549 cells (Figure 5a–d). Pathway inhibition studies revealed distinct internalization mechanisms where BPH uptake is predominantly governed by clathrin-mediated endocytosis with auxiliary contributions from macropinocytosis and caveolin pathways, whereas PBPH showed a substantial shift toward macropinocytosis-dominated uptake, consistent with protein-mediated cellular interactions (Figure 5a). For cellular internalization pathway inhibition studies of PBPH, cell viability and morphology were systematically evaluated to ensure reliable quantification of intra- and extracellular boron levels. Live/dead cell counts were correlated with representative bright-field images, confirming that most inhibitor conditions maintained acceptable viability with minimal morphological disruption (representative table and microscopic cellular images are reported in Figure S8a,b). Multiple independent data sets (≥5 replicates) were collected to minimize variability and ensure robustness in subsequent ICP-based quantification of boron uptake and trafficking.

**Figure 5:**
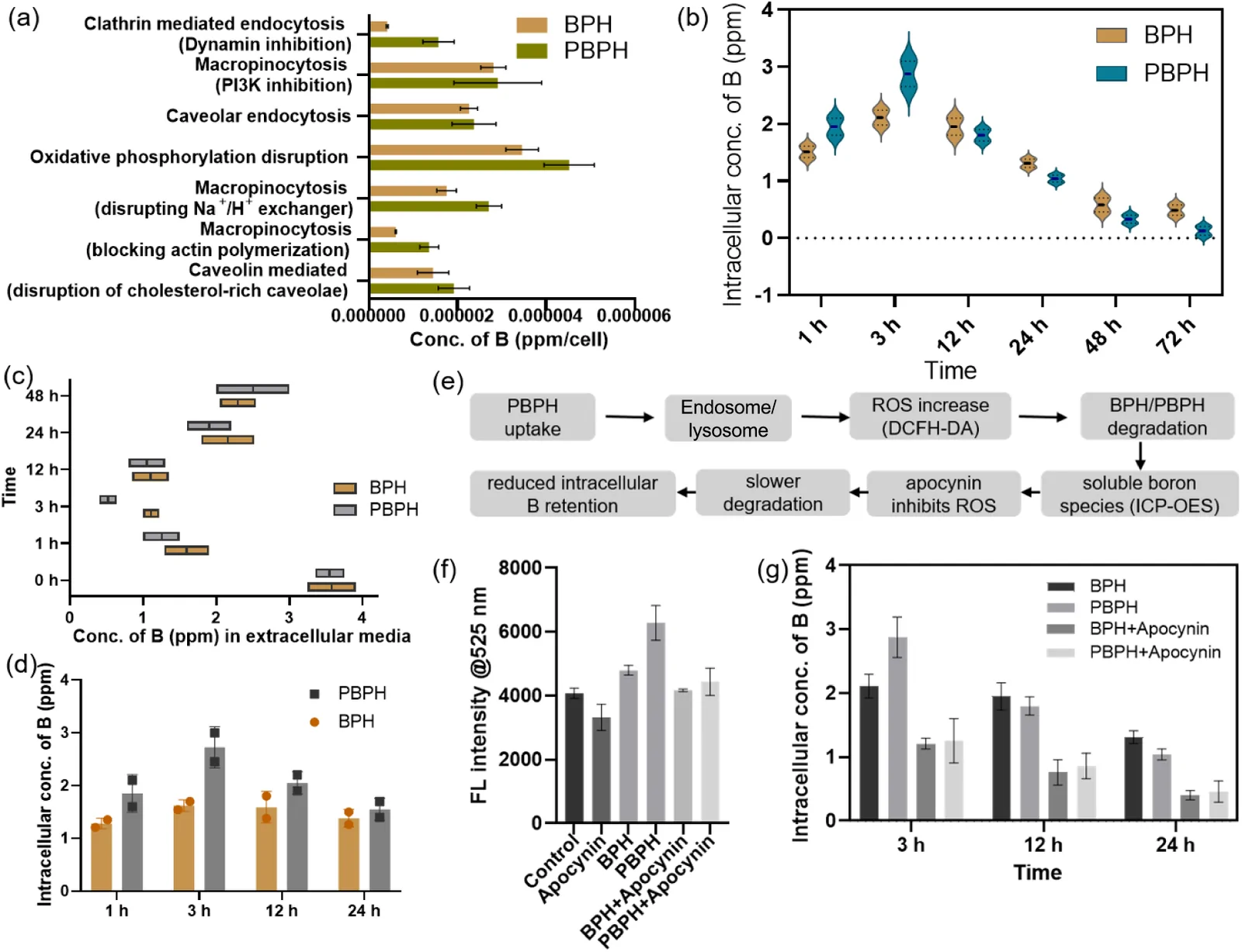
(a) Intracellular boron quantification following inhibition of endocytic pathways, showing clathrin-mediated uptake of BPH and macropinocytosis-driven uptake of PBPH. (b) Time-dependent intracellular boron levels (1–72 h) after exposure to BPH or PBPH. (c) Extracellular boron concentrations (0–48 h), reflecting release and degradation kinetics. (d) Intracellular boron levels in the presence of extracellular H_2_O_2_, demonstrating enhanced retention of PBPH relative to BPH. (e) Schematic for experimental chronology (f) Fluorescence from DCF shows differential fluorescence signals in the absence and presence of ROS-inhibitor. (g) Differential intracellular B concentrations for pristine BPH and PBPH with ROS inhibition.

Time-dependent ICP-MS/OES quantification revealed a biphasic intracellular boron profile (Figure 5b). During the initial 1–3 h, intracellular boron levels increased, reflecting rapid uptake, with a corresponding decrease in extracellular boron (Figure 5c), indicating efficient internalization.

A secondary phase (3–12 h) involved continued uptake and intracellular processing, leading to accumulation of degradation products. At 12–72 h, intracellular boron further increased and approached a plateau, while extracellular boron reached a quasi-steady state, suggesting a dynamic equilibrium between uptake, degradation, efflux, and reentry processes (Figure 5b,c).

Notably, PBPH exhibited higher intracellular boron retention under ROS-enriched conditions (H__2__O__2__ supplementation), indicating enhanced stability and sustained intracellular persistence relative to pristine BPH (Figure 5d). Despite this, PBPH also demonstrated faster intracellular processing kinetics, owing to improved dispersibility, increased effective surface area, and albumin-mediated uptake. Therefore, proteinization modulated both uptake pathways and intracellular degradation behavior, enabling efficient and controlled oxidative transformation.

### Intracellular ROS and Endolysosomal Processing Govern PBPH Degradation

To directly investigate the role of intracellular oxidative stress in PBPH degradation, ROS generation was quantified using CM-H2DCFDA. A consecutive intracellular ROS and boron concentration measurement mechanism has been illustrated through a schematic flowchart (Figure 5e). PBPH-treated A549 cells exhibited significantly higher dichlorofluorescein (DCF) fluorescence than untreated and pristine BPH-treated cells, indicating elevated intracellular ROS following PBPH internalization (Figure 5f). Apocynin pretreatment markedly reduced this signal, confirming effective suppression of oxidative activity.

To assess whether ROS influences PBPH processing, intracellular boron levels were measured by ICP-OES. PBPH-treated cells showed substantially higher boron accumulation than pristine BPH-treated cells (Figure 5g) due to better cellular internalization, whereas apocynin pretreatment significantly reduced intracellular boron levels for both pristine BPH and PBPH and the difference between BPH and PBPH are statistically not significant. These results indicate that intracellular ROS promotes PBPH degradation into smaller boron-containing species retained within cells, while ROS suppression slows degradation and facilitates the removal of intact or less-degraded PBPH, resulting in lower boron retention. Results demonstrate that intracellular ROS is a major contributor to PBPH degradation and support the proposed mechanism in which the HSA biointerfacial sheath protects borophene from premature extracellular oxidation while enabling environmentally responsive intracellular degradation. The enhanced intracellular degradation of PBPH is likely driven by the combined effects of ROS, endolysosomal acidification, and enzymatic remodeling of the HSA sheath, which progressively expose the pre-exfoliated borophene surface to oxidative transformation into soluble boronate species.

The direct ROS measurements further support our proposed environmentally responsive degradation mechanism. While the HSA biointerfacial sheath stabilizes borophene under extracellular physiological conditions, intracellular trafficking exposes PBPH to an oxidative endolysosomal environment that promotes degradation. Pharmacological suppression of intracellular oxidative activity using apocynin significantly reduced both ROS generation and intracellular boron accumulation, demonstrating that oxidative processing is an important determinant of PBPH intracellular fate. Because apocynin did not completely abolish PBPH processing, these findings further suggest that intracellular degradation likely arises from the combined effects of oxidative stress, acidic endolysosomal conditions, and enzymatic remodeling of the HSA interface rather than ROS alone.

### Hyperspectral Microscopy Reveals Intracellular Optical Signatures and Transformation Pathways

Hyperspectral microscopy (HSI) was employed to investigate the optical evolution,^51–53^ cellular localization, and time-dependent degradation of BPH and PBPH in extracellular and intracellular environments (Figure 6a). Pristine BPH exhibited narrow, well-defined spectral features dominated by high-intensity edge and basal-plane plasmonic resonances, characteristic of multilayered aggregates and crystalline integrity (Figure 6b,d,f). By contrast, HSA-functionalized PBPH exhibited broadened and red-shifted spectra with reduced scattering intensity, indicating enhanced exfoliation, improved dispersion, and modulation of the local dielectric environment via protein adsorption and surface coordination (Figure 6c,e,g).

**Figure 6:**
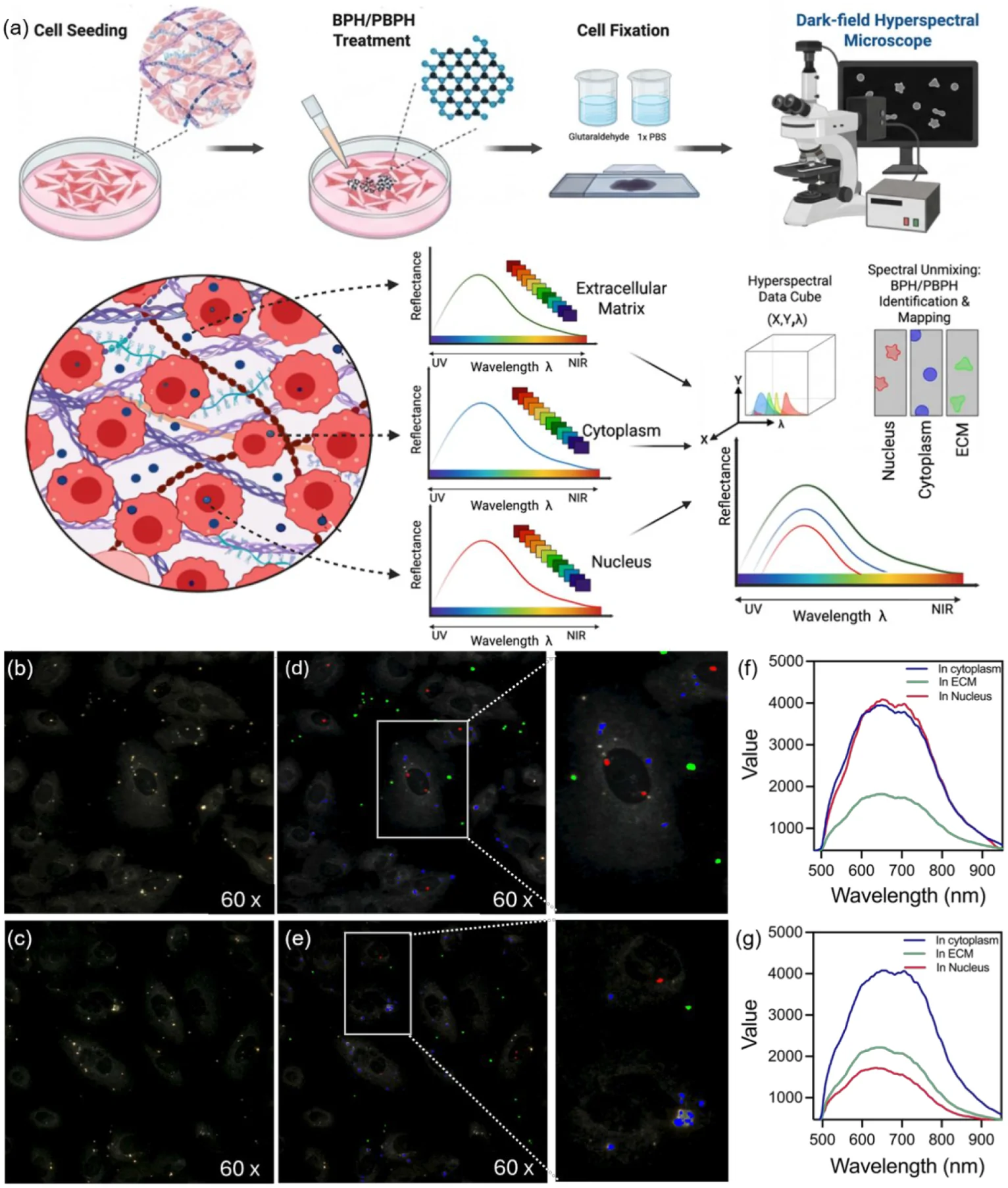
(a) Schematic of the dark-field hyperspectral imaging (HSI) workflow for mapping BPH/PBPH within cells. Representative dark-field images of A549 cells treated with (b) BPH and (c) PBPH. (d, e) Corresponding hyperspectral overlays showing spatial distribution of distinct spectral signatures. (f, g) Extracted spectra from the cytoplasm, ECM, and nucleus. BPH exhibits similar cytoplasmic and nuclear signatures with a distinct ECM profile, whereas PBPH shows divergence between cytoplasmic and nuclear spectra, consistent with intracellular interfacial restructuring and accelerated transformation following extracellular stabilization.

Dark-field HSI of fixed A549 cells, combined with reference spectral libraries derived from standardized H_2_O_2_-induced oxidation of BPH and PBPH in aqueous media, enabled pixel-wise classification of intact and transformed material states. Pristine BPH exhibited closely overlapping spectral profiles between cytoplasmic and nuclear compartments, with a spectrally distinct extracellular matrix (ECM) profile, suggesting that oxidative transformation occurs predominantly after cellular internalization rather than in the ECM (Figure 6f). By contrast, PBPH displayed nuclear spectra distinct from cytoplasmic profiles and closely resembled the ECM signature observed for oxidized BPH, indicating that efficient structural transformation once oxidative transformation is initiated despite initial extracellular stabilization imparted by HSA (Figure 6g). The ECM spectrum of PBPH appeared composite, reflecting coexistence of intact and partially transformed nanosheets and underscoring BIS-mediated spatiotemporal modulation of physicochemical transformations (Figure 6f,g).

### HRTEM Analysis of Intracellular Structural Degradation

Ultrastructural analysis of intracellular PBPH was performed using time-resolved TEM following standard fixation, staining, embedding, and ultrathin sectioning protocols^54,55^ to preserve cellular architecture (Figure 7a). A schematic (Figure 7b) illustrates PBPH nanosheets coated with an HSA-derived BIS, which modulates exposure to intracellular ROS and governs progressive surface transformation. Control A549 cells exhibited intact cytoplasmic morphology without electron-dense inclusions (Figures 7c, S9a). By contrast, PBPH-treated cells at 0.5 h displayed distinct electron-dense features within the cytoplasm at both micron and sub-200 nm scales (Figure 7d,e), confirming rapid cellular internalization.

**Figure 7:**
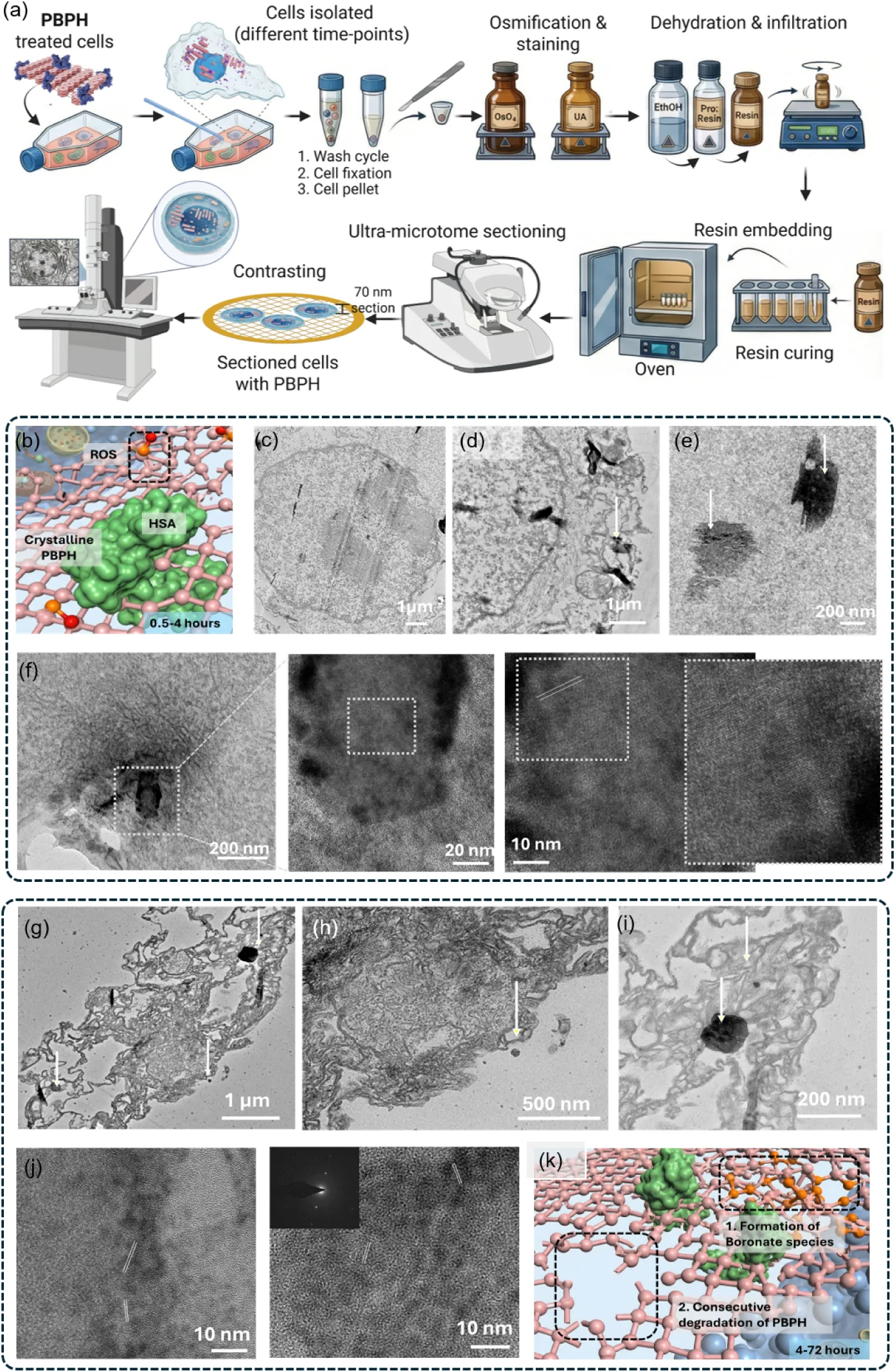
(a) Workflow for TEM-based ultrastructural analysis of PBPH-treated cells, including harvesting, fixation, staining, embedding, ultrathin sectioning, and imaging. (b) Schematic of crystalline PBPH under intracellular ROS-rich conditions, where the HSA biointerfacial sheath (BIS) modulates ROS exposure and early surface transformations (0.5–4 h). (c) Representative TEM image of untreated A549 cells. (d, e) TEM images of PBPH-treated cells at 0.5 h (1 μm and 200 nm scales); yellow arrows indicate intracellular PBPH. (f) Magnified views of PBPH-associated regions (white dashed box) showing nanosheet morphology and well-defined lattice fringes consistent with retained crystallinity. (g–i) TEM images of PBPH-treated cells at 4 h, with yellow arrows marking intracellular PBPH. (j) High-magnification image at 4 h showing nanosheet-like features and preserved crystalline domains. (k) Schematic of later intracellular evolution (4–72 h), illustrating progressive PBPH degradation and formation of boronate/oxidation products; dashed boxes indicate regions of structural loss.

At 4 h, BPH retained clearly defined lattice fringes, indicating retained crystalline integrity (Figure 7f,j). By contrast, PBPH showed partially lattice loss, suggesting early stage structural perturbation (Figure 7g–i). Extended incubation (4–72 h) demonstrated accelerated PBPH degradation, with progressive loss of lattice periodicity and formation of boronate oxidation products (Figures 7k, S9b). At 48–72 h, no detectable lattice fringes or intact particles remained in PBPH-treated cells, confirming complete structural degradation (Figure S9c). These observations demonstrate that although the HSA BIS initially stabilizes borophene and facilitates cellular uptake, it concurrently enables controlled and accelerated intracellular degradation under ROS-rich conditions, consistent with the dynamic processing behavior observed in complementary ICP-MS/OES analyses.

### Digital Holotomography Reveals 3D Spatial Distribution and Compartmentalization

Digital holotomography (DHT) enabled label-free 3D visualization of PBPH internalization in live cells using multiangle coherent illumination^56–59^ to generate holograms of BPH/PBPH-treated A549 cells (Figure 8a). Refractive index (RI)-induced phase shifts were computationally reconstructed employing inverse scattering algorithms to produce volumetric 3D RI maps, enabling quantitative *z*-axis mapping of intracellular PBPH distribution (Figure 8b,c). Maximum-intensity projection (MIP) renderings and corresponding 3D RI volume visualizations revealed PBPH as punctate, high-RI inclusions distributed throughout the cytoplasm, consistent with vesicular localization and endosomal trafficking (Figure 8d).

**Figure 8:**
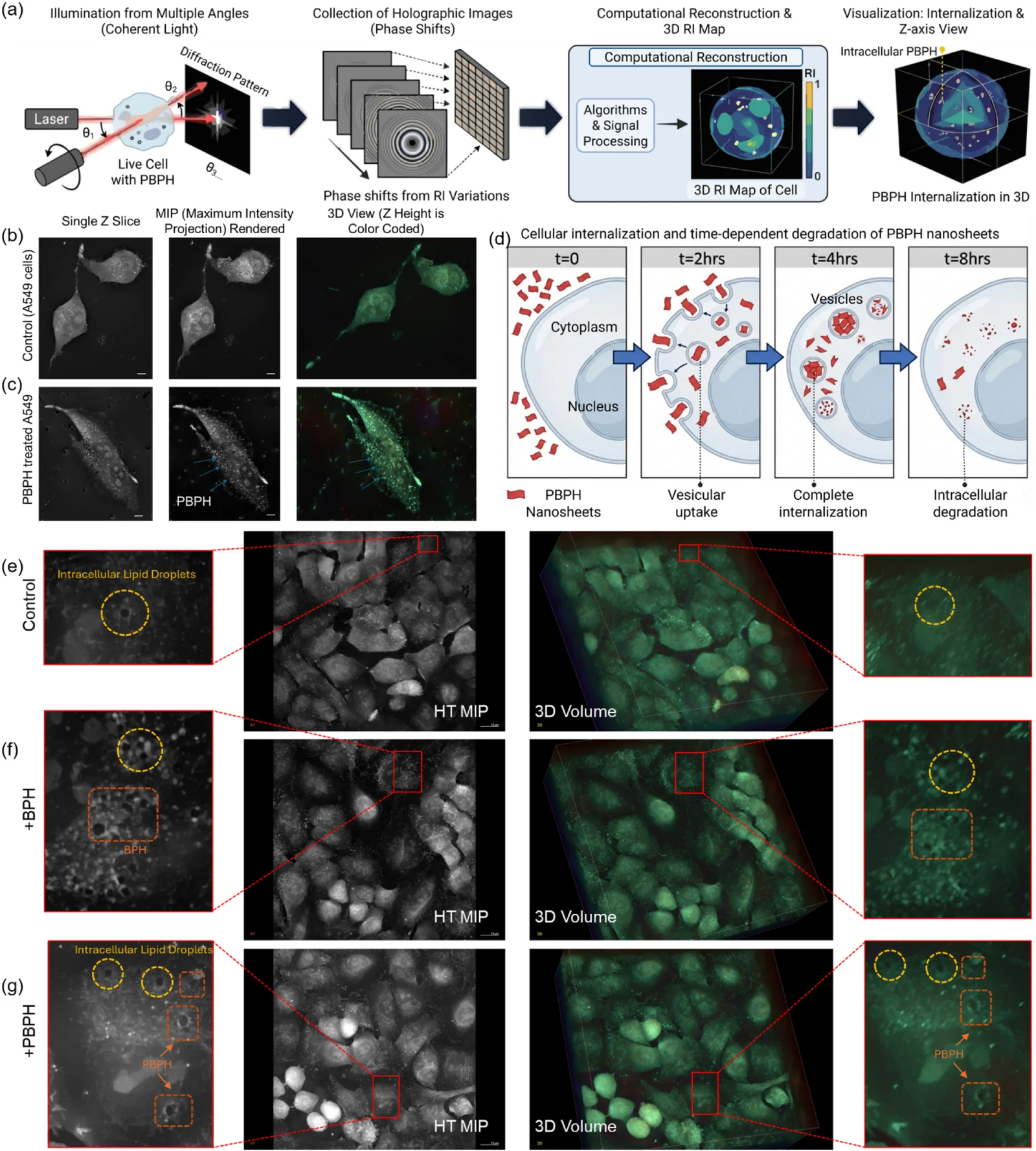
(a) Schematic of digital holotomography (DHT) for label-free 3D visualization of PBPH in live A549 cells. Multiangle coherent illumination generates holograms that are reconstructed into volumetric refractive index (RI) maps, enabling *z*-axis mapping of intracellular PBPH. (b, c) Representative maximum-intensity projections (left, middle) and corresponding 3D RI renderings showing PBPH as punctate high-RI inclusions distributed throughout the cytoplasm (arrows). (d) Schematic of intracellular PBPH processing (0–8 h), illustrating endosomal localization (∼2 h), accumulation and early transformation (∼4 h), and substantial degradation (∼8 h). (e) Control A549 cells imaged by DHT, showing endogenous high-RI lipid droplets. (f) Pristine BPH-treated cells displaying colocalization of BPH-associated high-RI inclusions with lipid droplets. (g) PBPH-treated cells showing intracellular PBPH-associated inclusions with similar spatial overlap and interaction patterns with lipid droplets, suggesting compartmental association during processing.

Time-resolved analysis demonstrated distinct spatiotemporal progression: at ∼2 h postexposure, PBPH localized predominantly in endosomes; by ∼4 h, accumulation and early structural transformation were observed; and by ∼8 h, significant cytoplasmic degradation occurred with dispersed low-RI signatures. Control A549 cells imaged via DHT displayed intrinsic high-RI intracellular lipid droplets in the absence of BPH exposure (Figure 8e). Pristine BPH-treated cells showed colocalization and apparent spatial convergence between BPH-associated high-RI inclusions and endogenous lipid droplets (Figure 8f), whereas PBPH-treated cells revealed intracellular PBPH-associated inclusions with spatial overlap and interaction patterns comparable to lipid droplets (Figure 8g), suggesting compartmental association during intracellular processing. These findings indicate that both BPH and PBPH undergo lipid droplet-mediated trafficking, with PBPH exhibiting enhanced interaction with lipophilic organelles likely attributed to HSA surface functionalization.

### Refractive Index-Based 3D Holotomography of Real-Time Internalization and Recycling

Refractive index-based DHT enabled longitudinal monitoring of PBPH internalization and intracellular processing in live A549 cells at 0, 6, 12, and 24 h (Figure 9a). Representative holotomographic RI images revealed distinct uptake and degradation kinetics between pristine BPH and PBPH over 0–8 h (Figure 9b,c). PBPH-treated cells exhibited more pronounced intracellular high-RI inclusions and progressive redistribution compared with BPH, indicating enhanced uptake and accelerated intracellular processing. Pseudocolor RI maps highlighted intracellular PBPH (green) relative to cellular structures (purple), with high-RI puncta corresponding to PBPH-containing vesicular compartments (Figure 9d). This label-free visualization enabled real-time tracking of subcellular localization and structural evolution throughout the degradation process.

**Figure 9:**
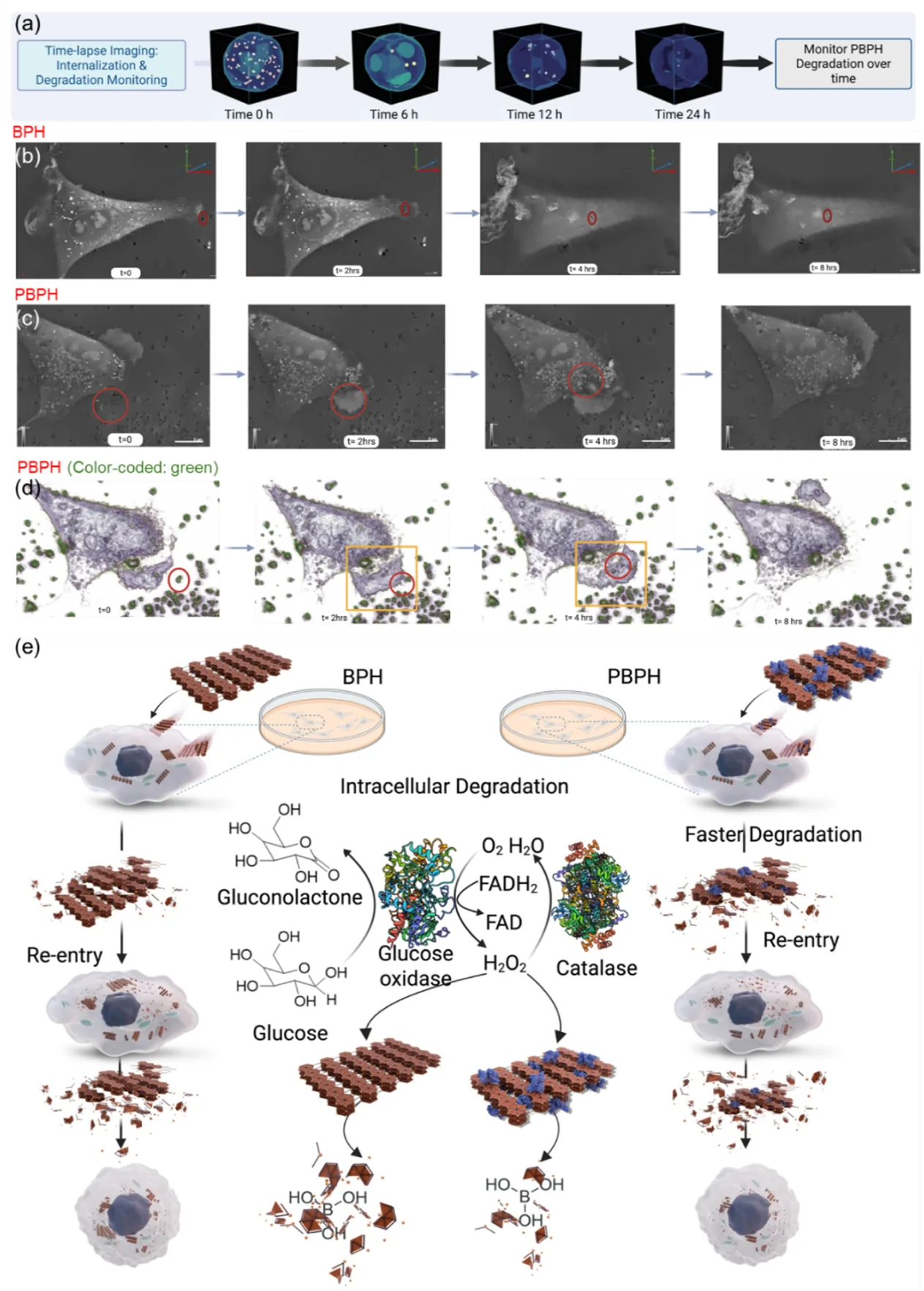
(a) Experimental workflow for longitudinal digital holotomography of live A549 cells, monitoring PBPH internalization and processing at 0, 6, 12, and 24 h. (b, c) Representative RI images of cells treated with (b) pristine BPH or (c) PBPH over 0–24 h. PBPH-treated cells show more pronounced high-RI inclusions and progressive redistribution, consistent with enhanced uptake and intracellular processing. (d) Pseudocolored RI maps highlighting intracellular PBPH (green) relative to cellular structures (purple), enabling label-free visualization of vesicular localization and structural evolution. (e) Proposed intracellular degradation mechanism of BPH and PBPH. Following endocytic uptake, oxidative transformation is driven by intracellular redox enzymes and H_2_O_2_ generation. PBPH undergoes accelerated degradation relative to BPH due to increased enzymatic accessibility and interfacial restructuring, producing boron-oxide/boronic acid species that are subsequently cleared or recycled.

The proposed mechanism of intracellular degradation involves oxidative transformation mediated by intracellular redox enzymes (Figure 9e). Following endocytic uptake, BPH underwent GOx-driven H_2_O_2_ generation and catalase-associated oxidative pathways, leading to gradual conversion to boronic acid species and subsequent reentry or clearance. PBPH exhibited accelerated degradation relative to pristine BPH, likely owing to increased enzymatic accessibility conferred by HSA functionalization and interfacial restructuring that promotes oxidative fragmentation. This resulted in faster formation of boron-oxide/boronic acid species and more efficient cellular clearance or reentry processes.

## Discussion

The present study shows that PBPH undergoes intracellular degradation to soluble boron species; however, the downstream metabolic fate and long-term biodistribution of these products remain to be determined. In physiological media, inorganic boron is expected to exist primarily as boric acid/borate, a highly soluble and readily cleared form that is eliminated mainly via renal excretion. Thus, the environmentally responsive degradation of PBPH may favor biological clearance by converting borophene into physiologically soluble boron species rather than persistent nanostructures.^9,10^ Nonetheless, dedicated in vivo pharmacokinetic, biodistribution, and long-term safety studies are still required to define clearance kinetics, tissue distribution, and any potential accumulation of PBPH-derived products, and these studies will be pursued in future work.

This study establishes a mechanistically grounded framework for protein-assisted surface exfoliation of 2D borophene, linking nanoscale material chemistry, biomolecular interactions, and intracellular oxidative biology. Spontaneous adsorption of HSA transforms BPH into a biocompatible, functionally adaptive PBPH complex via formation of a BIS, which not only induces spontaneous exfoliation into ultrathin sheets and suppresses aerial/serum-mediated oxidation but also modulates the surface electronic structure to attenuate electron deficiency and stabilize interfacial reactivity. PBPH exhibits efficient exfoliation into mono- and few-layer sheets, suppressed air- and serum-mediated oxidation, and substantially improved dispersion and colloidal stability in physiological media, enabling controlled integration of BPH into biological environments with stabilized yet responsive behavior.

Notably, the HSA-derived BIS extends beyond passivation of BPH by directing intracellular fate. Following receptor-mediated uptake and endocytosis, PBPH undergoes accelerated, spatially localized degradation driven by ROS such as H_2_O_2_ generated within endolysosomal compartments, effectively decoupling extracellular stabilization from intracellular disassembly. Multimodal characterization (HRTEM, cryo-TEM, SAXS/WAXS, XPS, SIMS, ICP MS/OES, holotomography, and HSI) demonstrates faster and more complete conversion of PBPH than pristine BPH into boronic acid derivatives, via progressive lattice fragmentation, crystallinity loss, and B–O bond formation, coupled to distinct spectral shifts that report exfoliation, oxidation, and intracellular breakdown in real time. Enzymatic pathways involving glucose oxidase, NADPH oxidases, and superoxide dismutase generate H_2_O_2_, while antioxidant enzymes such as catalase, myeloperoxidase, and glutathione peroxidase dynamically regulate local ROS levels, establishing an enzymatically tunable degradation axis. Complementary UV–vis spectroscopy, 3D refractive index imaging, and elemental analysis further quantify the enhanced degradation kinetics of PBPH relative to pristine BPH in ROS-rich environments.

Our results support a mechanistic model in which the HSA biointerfacial sheath (BIS) not only redirects borophene uptake from predominantly clathrin-mediated endocytosis to macropinocytosis but also regulates its environmentally responsive intracellular degradation. While the HSA BIS suppresses premature aerial and extracellular aqueous oxidation, preserving borophene integrity during transport, PBPH is trafficked into endolysosomal vesicles following internalization, as evidenced by TEM and holotomography. Within these compartments, acidic pH, lysosomal enzymes, and elevated ROS collectively remodel the HSA sheath, progressively exposing the borophene surface to oxidative transformation into soluble boronate species. The newly added DCFH-DA, apocynin, and ICP-OES experiments demonstrate that intracellular ROS is a major contributor to this process, whereas the incomplete inhibition by apocynin indicates that endolysosomal acidification and enzymatic remodeling also contribute to PBPH degradation. Although the precise molecular trafficking machinery remains to be elucidated, the combined experimental evidence supports a multifactorial endolysosomal degradation mechanism in which the engineered HSA BIS functions as a programmable regulator that suppresses extracellular oxidation while enabling controlled intracellular degradation.

## Conclusion

In conclusion, this protein-mediated surface exfoliation therefore represents a general strategy for engineering degradable 2D nanomaterials. The biointerfacial sheath stabilizes borophene externally while enabling ROS-regulated intracellular disassembly, a dual function difficult to achieve using conventional polymer or surfactant coatings. Minimal cytotoxicity despite enhanced ROS generation indicates that cellular antioxidant defenses can accommodate PBPH degradation without compromising viability, satisfying key safety criteria for translational use. Although the present work establishes the proof-of-concept for HSA-mediated regulation of borophene stability and intracellular degradation using a cancer cells, macrophages are expected to play critical roles in nanoparticle processing and clearance. Future studies will systematically investigate PBPH interactions with M1 and M2 tumor-associated macrophages, including phenotype-dependent uptake, intracellular degradation, immune modulation, and macrophage–tumor cell interactions to define the role of the biointerfacial sheath in the tumor immune microenvironment. These findings demonstrate that proteinized borophene surface engineering enables stabilization of labile 2D boron nanostructures, targeting via endogenous protein-recognition pathways, ROS-triggered conversion into soluble, excretable byproducts, and real-time traceability through optical and elemental signatures. These features highlight the broad potential for such systems as biodegradable, redox-responsive nanocarriers, theranostics, and transient imaging agents, while underscoring proteinized surface exfoliation as a versatile route to couple stability, transformation and clearance in reactive 3D materials.

## Methods

### Atomic Force Microscopy

AFM was performed utilizing a model 5600Ls atomic force microscope (Bruker Nano, USA). The analyses were performed in tapping mode at varying sizes, employing phase contrast and height modes. The raw images were processed using Nanoscope Analysis 3.0 software package.

### X-ray Photoelectron Spectroscopy

The samples were drop-casted multiple times and vacuum-dried each time to form a thick layer. XPS experiments were performed using a Physical Electronics VersaProbe III instrument equipped with a monochromatic Al Kα X-ray source (*h*ν = 1486.6 eV) and a concentric hemispherical analyzer. Charge neutralization was performed employing both low-energy electrons (<5 eV) and argon ions. The binding energy axis was calibrated using sputter-cleaned Cu (Cu 2p_3/2_ = 932.62 eV, Cu 3p_3/2_ = 75.1 eV) and Au foils (Au 4f_7/2_ = 83.96 eV). Peaks were charge referenced to the CH_*x*_ band in the carbon 1s spectra at 284.8 eV. Spectra were acquired at a takeoff angle of 45° with respect to the sample surface plane, resulting in a typical sampling depth of 3–6 nm (95% of the signal originated from this depth or shallower). Quantification employed instrumental relative sensitivity factors (RSFs) that represent the X-ray cross section and inelastic mean free path of the electrons. The analysis size was ∼200 μm in diameter. Data analysis was performed using CasaXPS software.

### TEM Characterization

TEM characterization procedure followed standard protocols. A 3.5 μL aliquot of nanoparticle (NP) dispersion was drop cast onto carbon-coated copper grids and blotted to dryness. Imaging, selected area electron diffraction, and energy-dispersive X-ray spectroscopy (EDS) was performed on a Talos F200X (Thermo Fisher Scientific) TEM operated at 200 kV, equipped with a Ceta CMOS CCD camera and four in-column SDD Super-X detectors. For EDS, elemental maps were acquired for approximately 5 min (beam current = 0.12 nA) and analyzed using Velox software. Additional image analysis was performed using ImageJ.

### Scanning Electron Microscopy

Scanning electron microscopy was employed to characterize the surface morphology of borophene (BPH) and HSA-conjugated borophene (PBPH), and to confirm successful HSA functionalization. Imaging was performed on a Thermo Scientific Verios G4 field-emission scanning electron microscope under high-resolution conditions to visualize HSA coverage on the BPH sheets.

### High-Performance Liquid Chromatography–Mass Spectrometry

160 mM boric acid (aq) was diluted to 1 mM boric acid in 50% (v/v) LC-MS grade methanol and 0.3% (v/v) Ammonium Hydroxide. The ammonium hydroxide was added to make the solution basic (∼pH 9.5), which facilitates boric acid ionization. The solution was directly injected onto the Thermo Q-exactive Mass Spectrometer and was ionized via electrospray ionization (ESI). Mass spectra were acquired over an *m*/*z* range of 50–500 in negative ion mode.

### Cryogenic Transmission Electron Microscopy

For cryogenic transmission electrons, samples were vitrified using a Vitrobot Mark IV system (Thermo Scientific) on Quantifoil R 2/2 copper grids (200 mesh; Quantifoil, Germany). Prior to sample application, grids were glow-discharged at 15 mA for 30 s utilizing a PELCO easiGlow system. For each grid, 3.5 μL of the nanoparticle suspension was deposited under controlled conditions of 4 °C and 100% relative humidity, followed by blotting for 4 s. Cryo-electron microscopy data were collected on a Titan Krios G3 microscope (Thermo Scientific) equipped with a Falcon 4 direct electron detector. Imaging was performed at an accelerating voltage of 300 keV, using a nominal magnification of 37,000×, spot size of 6, and C2 aperture, yielding a calibrated pixel size of 1.8 Å.

### XRD

Powder X-ray diffraction was conducted on an Empyrean III diffractometer (Malvern Panalytical Inc.) equipped with a Cu Kα source. Diffractograms were collected over 2θ values ranging from 5° to 70°, with a step size of 0.026°.

### ICP-MS Uptake Kinetics

To examine NPs internalization, A549 cells were seeded in duplicate 6-well plates at 1.5 × 10^5^ cells/well and incubated overnight. Treatment solutions were added and incubated for 2, 4, or 6 h. At each time point, media were removed, and wells were washed three times with PBS 1× to remove extracellular particles. Cells were detached with 1 mL/well Trypsin-EDTA (0.25%) for 3 min, pelleted, resuspended in 0.5 mL of Milli-Q water, and counted. Cell suspensions were transferred to glass vials for acid digestion using 0.5 mL ICP-grade HNO_3_ and left loosely capped in a fume hood for 72 h. Samples were then diluted to 10 mL with Milli-Q water and analyzed via ICP-MS. Stock treatment solutions were digested in parallel.

### Cell Culture

A549 (lung cancer cell line) was obtained from the American Type Culture Collection (ATCC; MA, USA). The cells were cultured at 37 °C in F-12K medium with 10% (v/v) fetal bovine serum (GIBCO) and 1% (v/v) penstrep (Invitrogen) as supplements. The cells were retained for further use after trypsinization with 0.25% trypsin-EDTA and grown in T75 tissue culture flasks (Nunc).

### Hyperspectral Study

Hyperspectral imaging was performed at a spectral resolution of 1.5 nm over the visible to near-infrared wavelength range (400–1000 nm) by a CytoViva spectrometer to detect minute pixel-to-pixel spectral differences. A quartz halogen aluminum reflector lamp with 75% power was used as the illumination source. The hyperspectral image was captured using a CCD camera, and the data were processed using ENVI software.

### Molecular Dynamics Simulations

All-atom molecular dynamics simulations were performed to investigate HSA–BPH interactions. The BPH sheet was generated using a custom Python script based on a validated crystal structure constructed in VESTA, while the protein structure (PDB ID: 1AO6) was obtained from the RCSB Protein Data Bank and used as the initial conformation. The initial configurations of the BPH–protein assemblies were constructed using PACKMOL. Simulations were conducted in GROMACS 2025.2 (MPI-enabled) using the OPLS-AA force field. Energy minimization was performed using the steepest descent algorithm until the maximum force was <100 kJ·mol^–1^·nm^–1^. Equilibration proceeded in two stages: a 1 ns NVT ensemble at 300 K using the V-rescale thermostat (τ = 1 ps) with positional restraints applied to BPH and protein atoms unrestrained, followed by a 5 ns NPT ensemble at 1 bar using the Parrinello–Rahman barostat (τ = 5 ps) with restraints removed unless otherwise specified. A 2 fs integration time step was used throughout, and short-range electrostatic and van der Waals interactions were treated with a 1.0 nm cutoff.

### Isothermal Calorimetry

ITC measurements were done in water at 30 °C on a Microcal and analyzed in NanoAnalyzer. We used 350 μL of 0.1 mg/mL Borophene and 100 μL of the 100 μM Albumin. We manually titrated 2 μL of 24 Albumin injections. For control serum and PEG experiments same temperature, concentration and injections were used.

### Holotomography Imaging of BPH- and PBPH-Treated Cells

#### Imaging Platform

Three-dimensional refractive index (RI) tomograms were acquired using a low-coherence holotomography system (HT-X1, Tomocube Inc.). The system comprised an LED illumination source (λ ≈ 450 nm) integrated with a digital micromirror device (DMD) to generate multiple optimized illumination patterns for RI reconstruction. Transmitted light from the sample was collected using a 4-f optical system equipped with a high numerical aperture (NA 0.95) 40× objective and detected employing a CMOS camera. Axial scanning was performed over ∼140 μm with a step size of ∼947 nm, enabling volumetric acquisition. The resulting intensity stacks were computationally reconstructed into 3D RI maps via deconvolution with system-specific optical transfer functions. The system provides lateral and axial resolutions of ∼155 nm and ∼947 nm, respectively. All imaging operations were controlled using TomoStudio X software, and a stage-top incubation chamber maintained physiological temperature, humidity, and CO_2_ levels during live-cell imaging.

#### Sample Preparation

A549 cells were seeded onto glass-bottom imaging dishes (#1.5H thickness compatible) and cultured under standard conditions. Cells were treated with BPH or PBPH dispersions at defined concentrations and incubated for specified time points prior to imaging. Prior to acquisition, culture media were replaced with fresh imaging medium, and dishes were directly transferred to the holotomography chamber without fixation to enable label-free, live-cell imaging.

#### Image Acquisition and Reconstruction

For each field of view, multiple transmitted intensity images were recorded under optimized Köhler illumination patterns generated via the DMD. These data sets were reconstructed into quantitative 3D RI distributions using inverse deconvolution algorithms based on theoretical point spread functions. In addition to RI maps, single-channel bright-field (scBF) images were simultaneously acquired for structural reference. Wide-field imaging was achieved by tiling adjacent fields of view (∼227 × 227 μm) with ∼23 μm overlap and subsequent stitching.

#### Image Processing and Analysis

All-in-focus RI projections were generated from 3D stacks using a normalized variance-based algorithm to extract the optimal focal plane across axial sections. Time-resolved RI maps were analyzed to track intracellular distribution, accumulation, and degradation of BPH and PBPH within cytoplasmic and nuclear regions. Quantitative analysis of RI contrast enabled label-free discrimination of nanomaterial-associated regions and their progressive transformation under intracellular conditions.

## Supplementary Material

























## Data Availability

The data that support the findings of this study are available in the Supporting Information of this article.

## Notes

Concentration-dependent oxidative degradation of borophene; spectroscopic and structural characterization of pristine and proteinized borophene; biomolecule-mediated stabilization under oxidative conditions; cellular viability and internalization pathway studies in A549 cells; time-resolved intracellular analysis of proteinized borophene (Figures S1–S9) (PDF)
BPH 16 h timelapse fast (MP4)
Figure 8b: BPH 16 h timelapse slow with Z-axis (MP4)
Figure 8c: PBPH scavenging and degradation, 12 h timelapse through Z-axis (MP4)
Figure 8d: MIP-rendered PBPH (color-coded) scavenge and macropinocytosis, 16 h timelapse (MP4)
MIP-rendered BPH, 16 h timelapse 1 (MP4)
MIP-rendered intra- and extracellular PBPH (differently color-coded) (MP4)
PBPH 12 h timelapse differentiating lipid droplets and BPH particles through Z-axis (MP4)
PBPH 2 h timelapse (MP4)
PBPH 4 h timelapse, cell scavenging and exocytosis (MP4)
PBPH degradation, 8 h timelapse through Z-axis (MP4)
PBPH degradation after macropinocytosis, 8 h timelapse (MP4)
