# Supplementary material for: Proteinized Surface Remodeling Decouples Environmental Stability from Intracellular Degradation in Oxidation-Prone 2D Nanoborophene
Source: ACS Nano. 2026 Sep 9;20(37):25674–92. doi: 10.1021/acsnano.6c11186 (PMC13602918; doi:10.1021/acsnano.6c11186)
Supplement: Supplementary file 1 [file nn6c11186_si_001.pdf]

Proteinized Surface Remodeling Decouples Environmental Stability From Intracellular Degradation in Oxidation-Prone 2D Nano-Borophene

Pranay Saha[a,b†], Shraddha Krishnakumar[c†], Teresa Aditya[a], Nada Maher[a‡], Aysenur Yardim[a,h‡], Oguzhan Colak[a‡], David Skrodzki[d‡], Sung Hyun Cho[b], Xu Feng[i], Dongjun Shin[j], Dipanjan Pan [a,b,c,d,e,f,g*]

[a]Department of Nuclear Engineering, The Pennsylvania State University, University Park, PA 16802, USA

[b]Huck Institutes of the Life Sciences, 101 Huck Life Sciences Building, University Park, PA 16802, USA

[c]Department of Biomedical Engineering, The Pennsylvania State University, University Park, PA 16802, USA

[d]Department of Materials Science and Engineering, The Pennsylvania State University, University Park, PA 16802, USA

[e]Department of Chemistry, The Pennsylvania State University, University Park, PA 16802, USA

[f]Materials Research Institute, The Pennsylvania State University, University Park, PA 16802, USA

[g]Center for Infectious Disease Dynamics, The Pennsylvania State University, University Park, PA 16802, USA

[h]Department of Bioengineering, Institute of Natural Sciences, Ege University, Izmir, Türkiye

[i]Surface Analysis Facility, University of Delaware, Newark, DE 19716, USA

[j]Tomocube Inc., San Diego, CA, USA

[*]Email: dipanjan@psu.edu

[†,‡]Equal contribution

Supporting Information

Figure S1: Concentration dependent oxidative degradation of borophene monitored by UV via spectroscopy

Figure S2: Raman and FTIR spectra of pristine borophene and H2O2-treated borophene

Figure S3: Biomolecule screening for stabilization of borophene under oxidative conditions.

Figure S4: Solid state 11B NMR of proteinized borophene.

Figure S5: HRTEM and Cryo-TEM images of multilayered borophene.

Figure S6: SAXS/WAXS profiles of borophene and proteinized borophene under native and oxidative conditions

Figure S7: X-ray diffraction patterns of pristine borophene and proteinized borophene.

Figure S8: Assessment of A549 cell viability and morphology following pharmacological inhibition of proteinized borophene internalization pathways.

Figure S9: Time resolved HRTEM analysis of intracellular proteinized borophene in A549 cells.

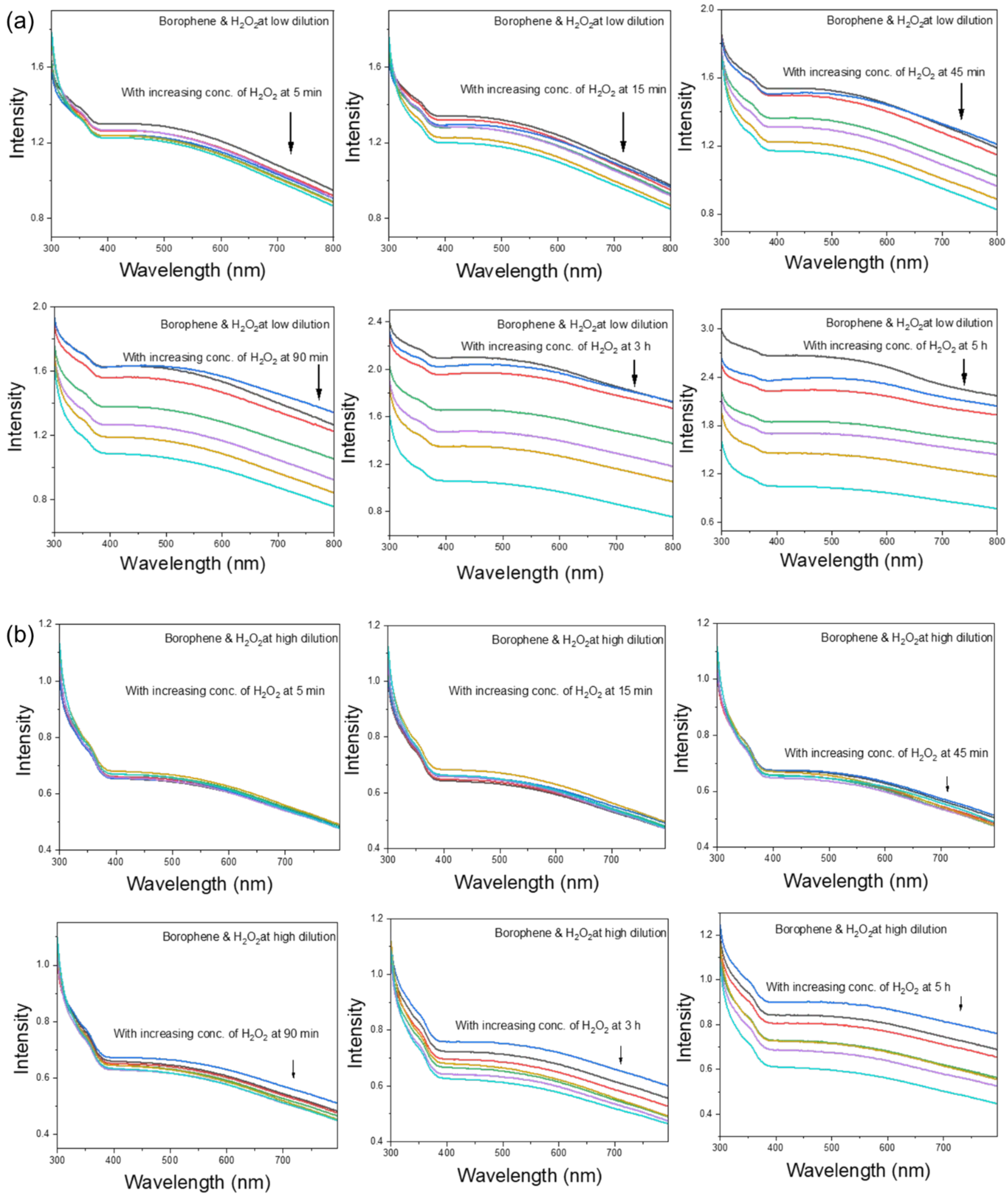

Figure S1. Concentration-dependent oxidative degradation of borophene monitored by UV–vis spectroscopy exhibited (a) Time-resolved spectra of borophene exposed to higher H_2O_2 concentrations showing rapid attenuation of absorbance and increased optical transparency,

indicative of accelerated degradation and conversion to soluble borate species. (b) Corresponding spectra at lower H_2O_2 concentrations exhibiting slower absorbance decay, reflecting reduced degradation kinetics and gradual transformation of borophene into borate products.

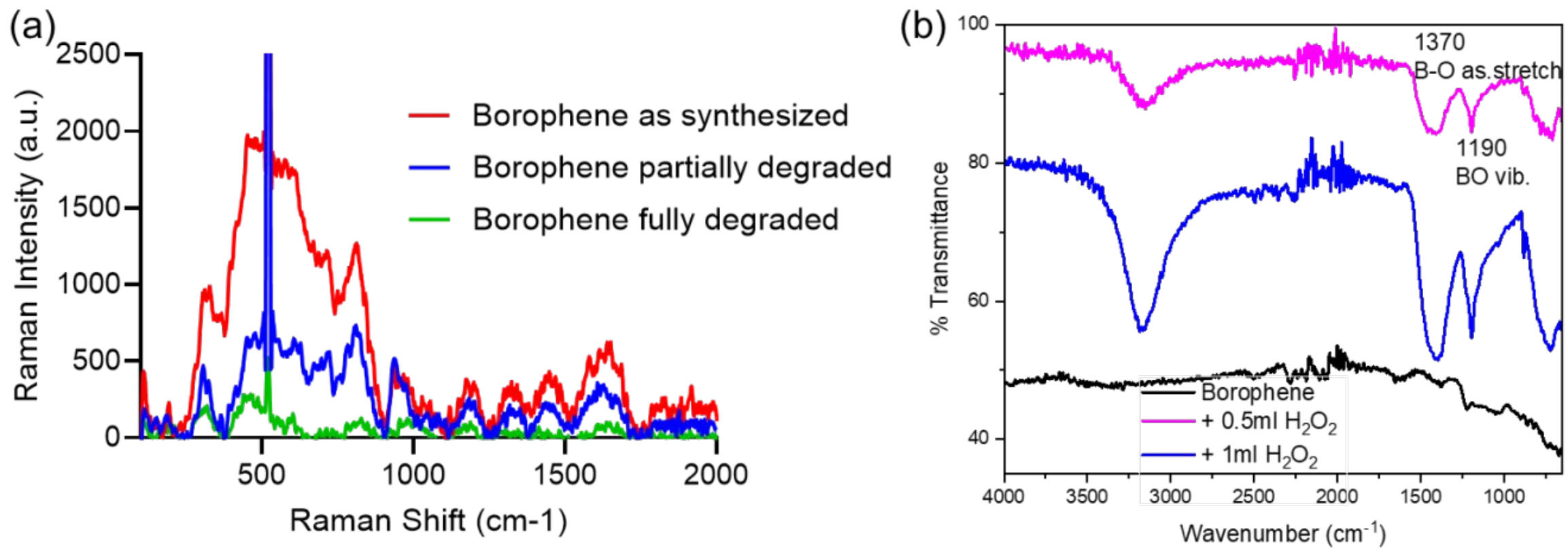

Figure S2. (a) Raman spectra of pristine and H_2O_2-treated BPH showing progressive attenuation of borophene vibrational modes, indicative of lattice disruption and structural degradation. (b) FTIR spectra of BPH before and after H_2O_2 exposure, highlighting the emergence of B–O stretching (~1350–1450 cm^{-1}) and broad O–H bands (~3200–3500 cm^{-1}), confirming oxidative conversion to boronic/borate and hydrated oxide species.

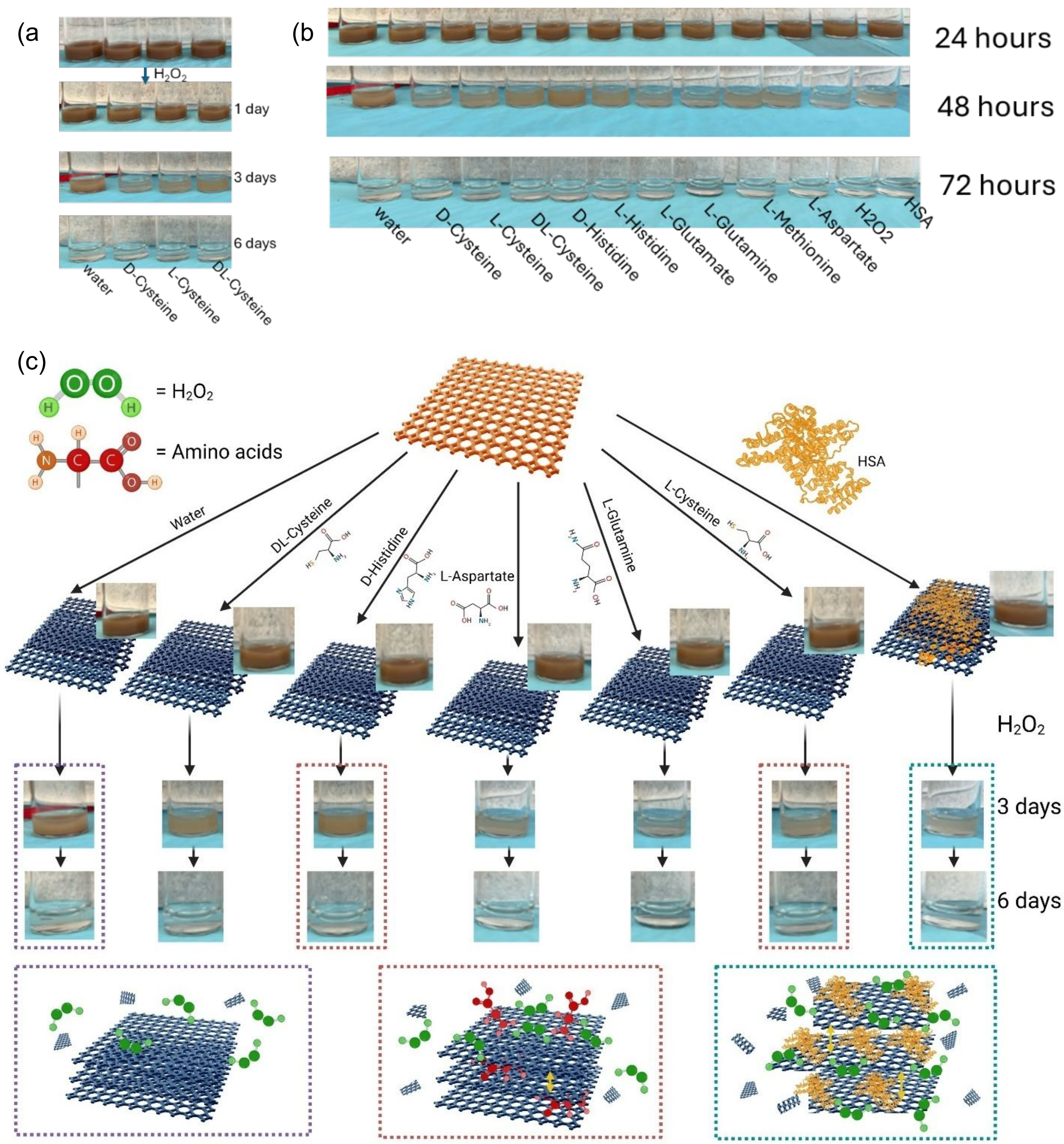

Figure S3. Biomolecule screening for stabilization of borophene under oxidative conditions (a,b) Photographic images showing time-dependent degradation of borophene dispersions in water and

in the presence of various biomolecules under H_2O_2 exposure, highlighting rapid loss of turbidity and increased transparency due to oxidative conversion into soluble borate species. (c) Schematic illustrating the interaction of borophene with different amino acids (e.g., cysteine, histidine, aspartate, glutamine) and HSA under ROS-rich conditions. Small biomolecules provide limited surface protection, resulting in accelerated oxidation and fragmentation, whereas HSA forms a conformal biointerfacial sheath that effectively shields the borophene surface. The bottom panels depict corresponding experimental observations over 3–6 days and mechanistic representations, demonstrating that HSA uniquely preserves structural integrity by limiting ROS accessibility through multivalent interactions and dynamic surface coverage.

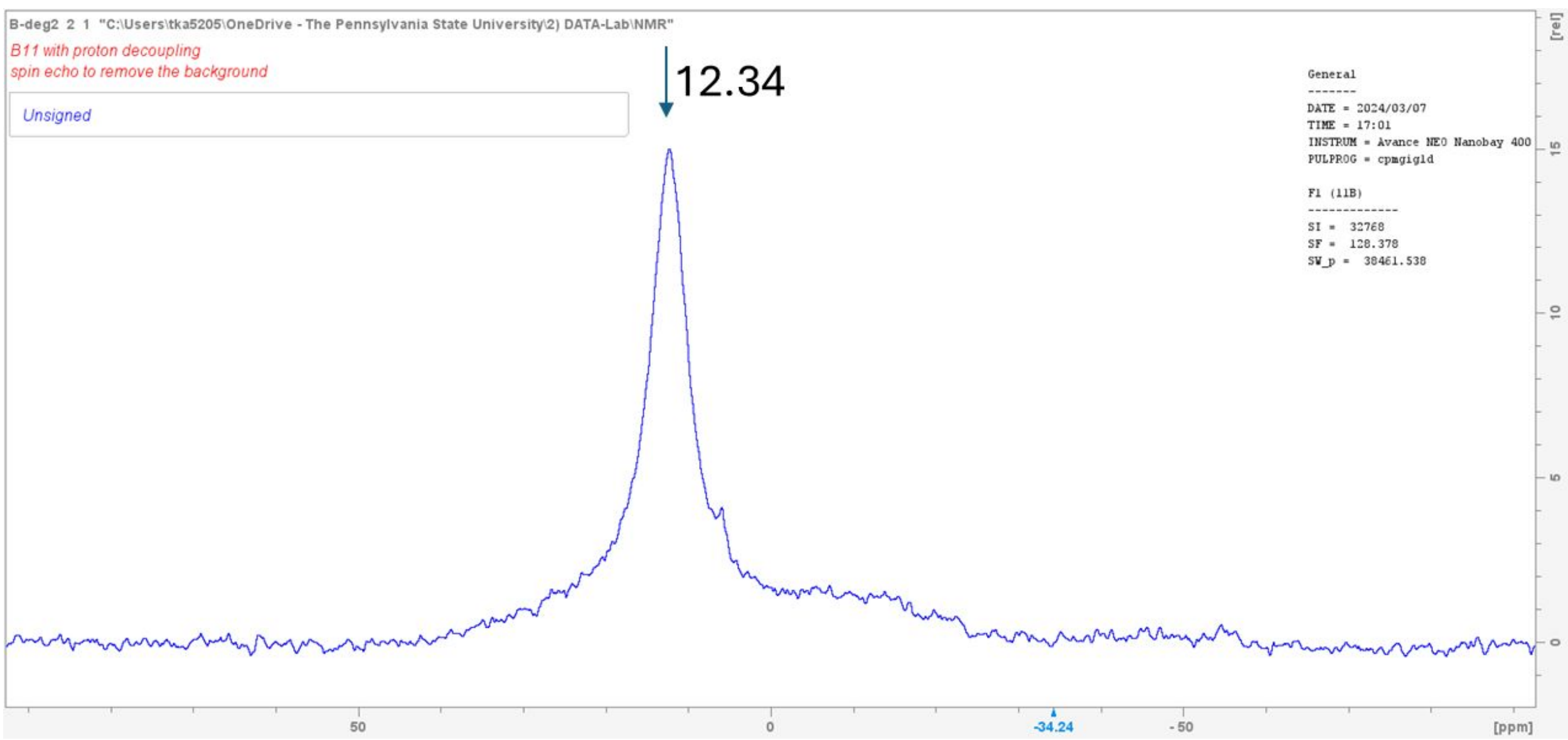

Figure S4. Solid-state ^{11}B NMR of PBPH displays a broad resonance centered at ~12.3 ppm, characteristic of boron in a predominantly B–O coordinated environment, confirming preservation of the boron framework with surface oxidation/interfacial modification following HSA association

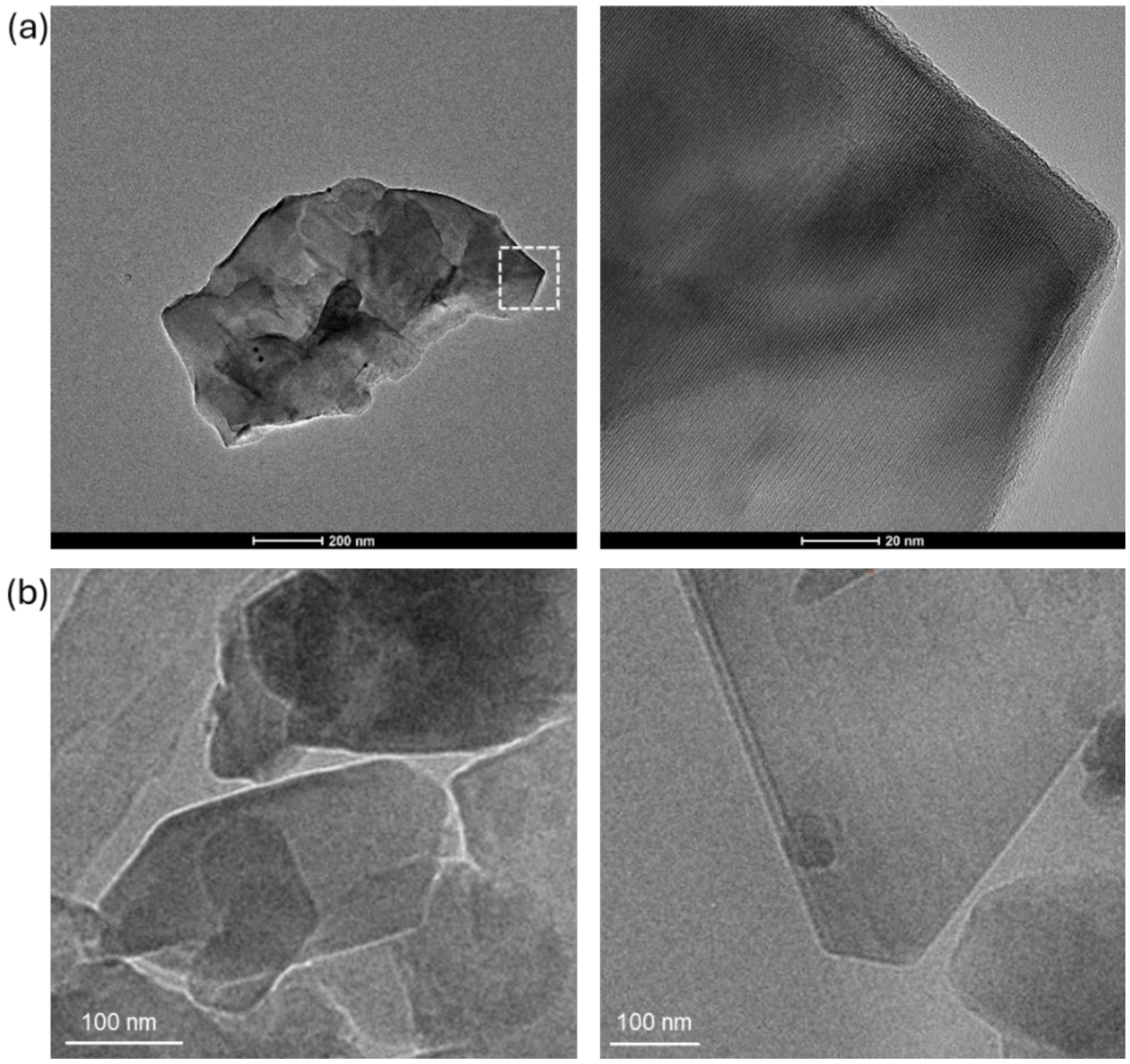

Figure S5. (a) HRTEM images reveal multilayered borophene exhibiting characteristic crystalline lattice fringes (box zoomed in). (b) Cryo-TEM retains similar multilayer arrangement and crystallinity in aqueous media

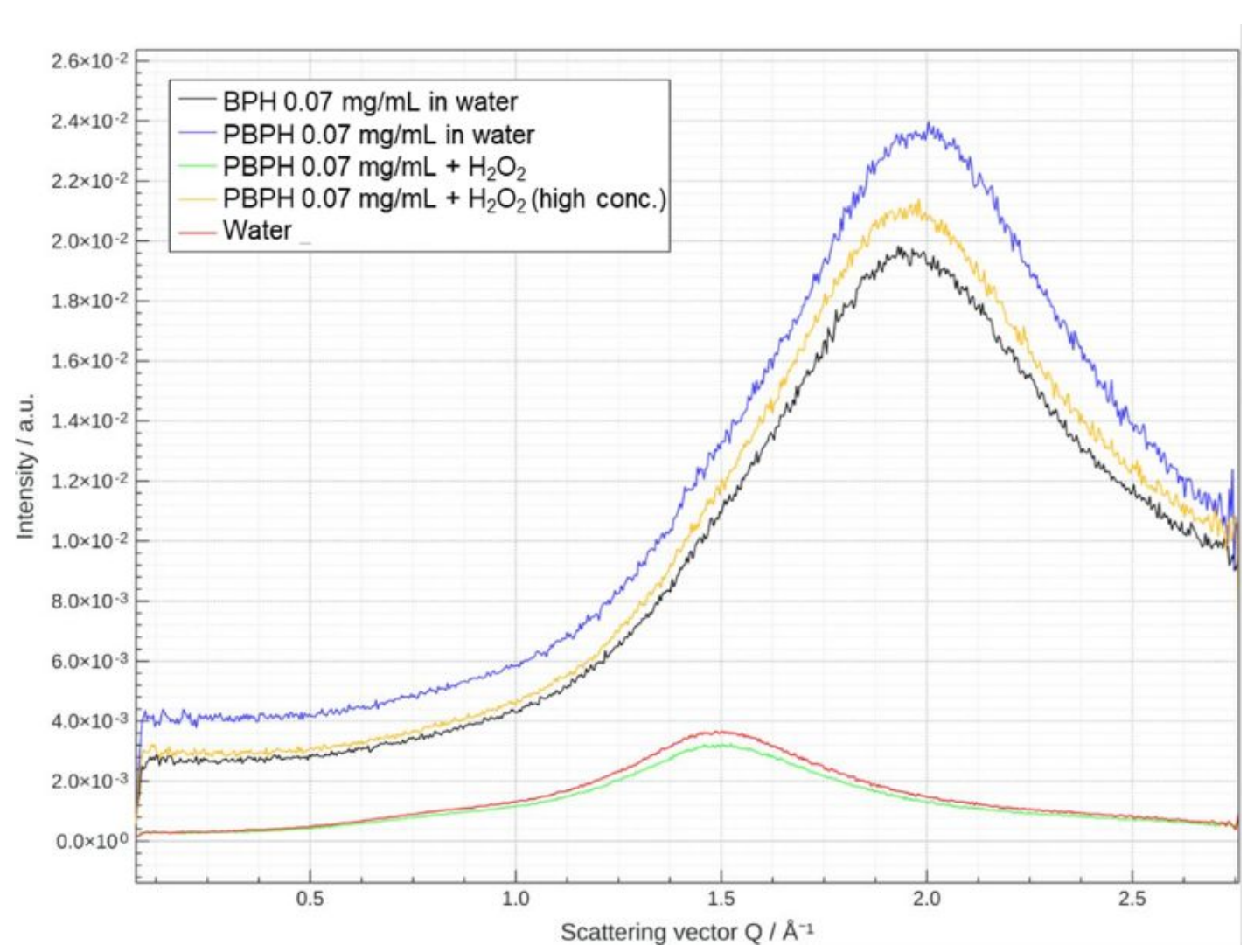

Figure S6. SAXS/WAXS profiles of borophene (BPH) and proteinized borophene (PBPH) under native and oxidative conditions exhibits a broad scattering maximum centered at $q \approx 1.9$ Å^{-1}, corresponding to a characteristic d-spacing of ~2.9 Å, is observed for both BPH and PBPH, indicating preserved short-range structural ordering upon protein adsorption. Upon exposure to H_2O_2, PBPH shows a progressive decrease in peak intensity and structural definition, consistent with oxidative degradation and loss of local atomic correlations. DI water is shown as background control.

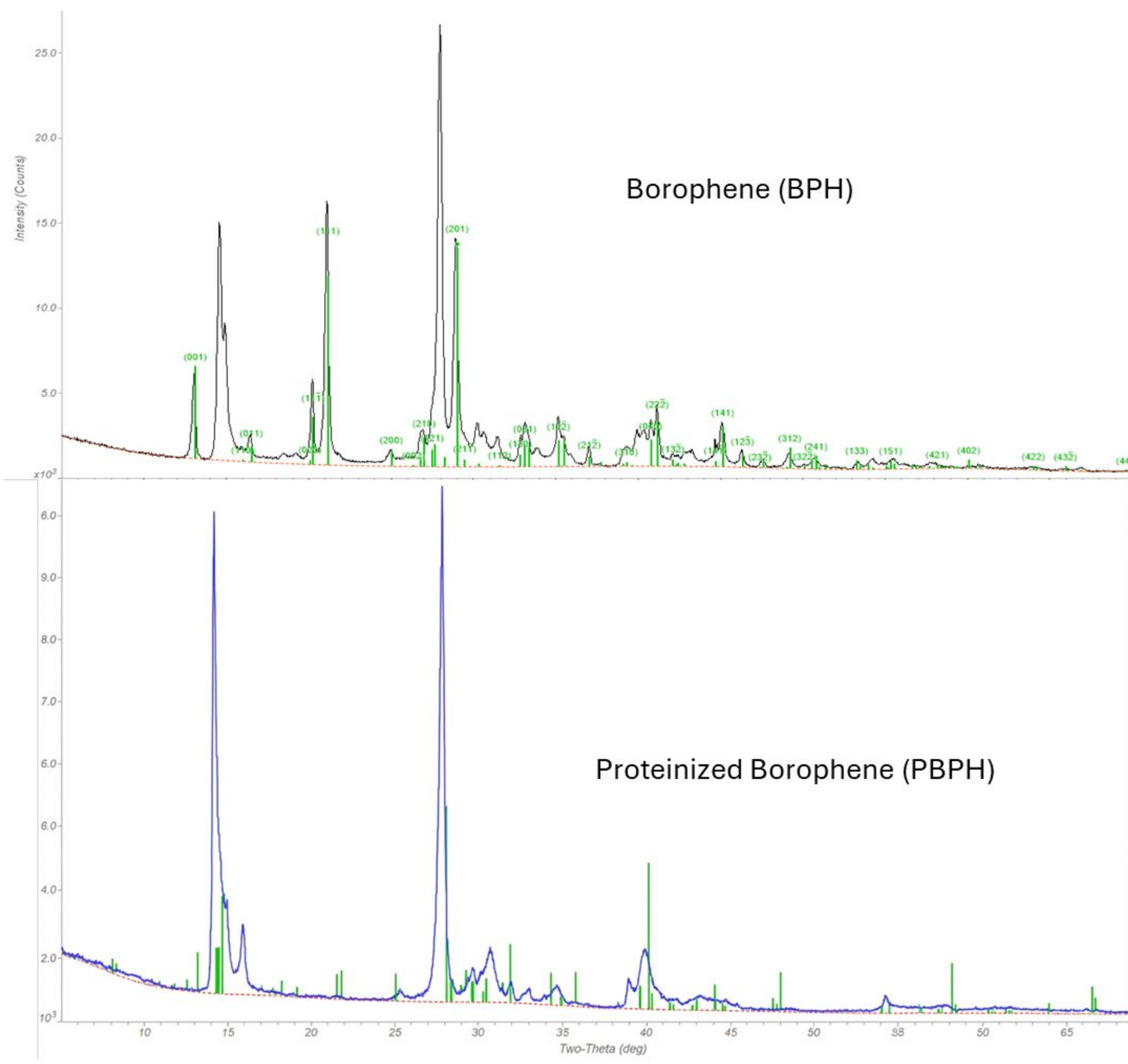

Figure S7. Full X-ray diffraction (XRD) patterns of pristine BPH and PBPH stacked vertically.

(a)

	Live cells	dead cell %
Control	5.69×10^{5}/mL	2% dead
Wortmanin	5.75×10^{5}/mL	9% dead
(EIPA), low conc.	9.09×10^{5}/mL	6% dead
(EIPA), high conc.	8.97×10^{5}/mL	12% dead
Cytochalasin D, low conc.	3.36×10^{6}/mL	64% dead
Cytochalasin D, high conc.	4.12×10^{6}/mL	65% dead
M β CD	1.04×10^{6}/mL	15% dead
Dynasore	1.24×10^{6}/mL	2% dead
SA+2DG, high conc.	4.52×10^{5}/mL	29% dead
SA+2DG, low conc.	8.15×10^{5}/mL	16% dead
Nystatin, high conc.	6.80×10^{5}/mL	11% dead
Nystatin, low conc.	9.44×10^{5}/mL	9% dead

(b)

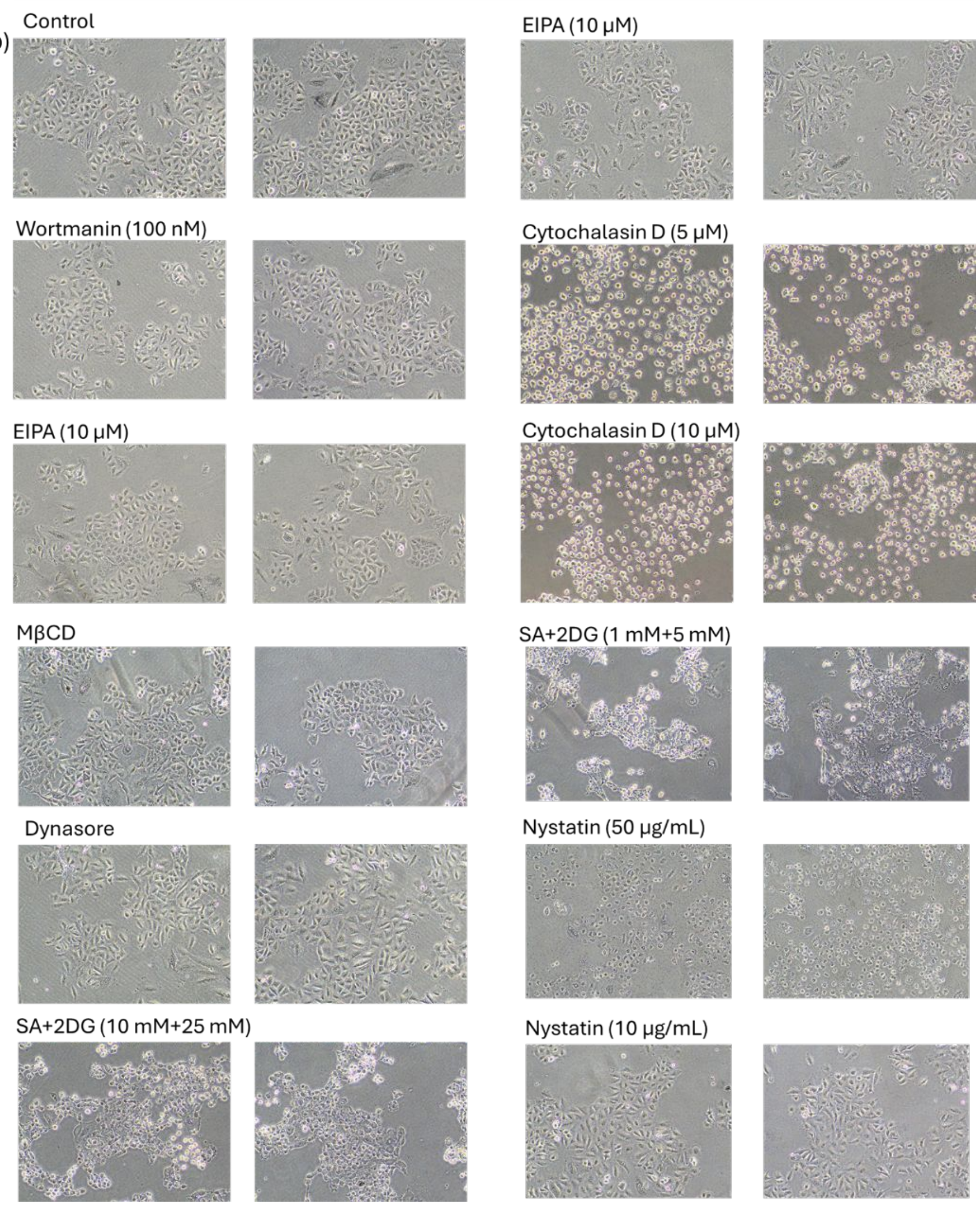

Figure S8. (a) Quantitative analysis of live cell counts and corresponding percentage of dead cells under different pharmacological inhibitors used to probe PBPH internalization pathways. Most conditions maintained low cytotoxicity, while cytochalasin D treatments showed elevated cell death. (b) Representative bright-field microscopy images of A549 cells under each inhibition condition, illustrating preserved cellular morphology in most treatments and noticeable morphological alterations in highly cytotoxic conditions.

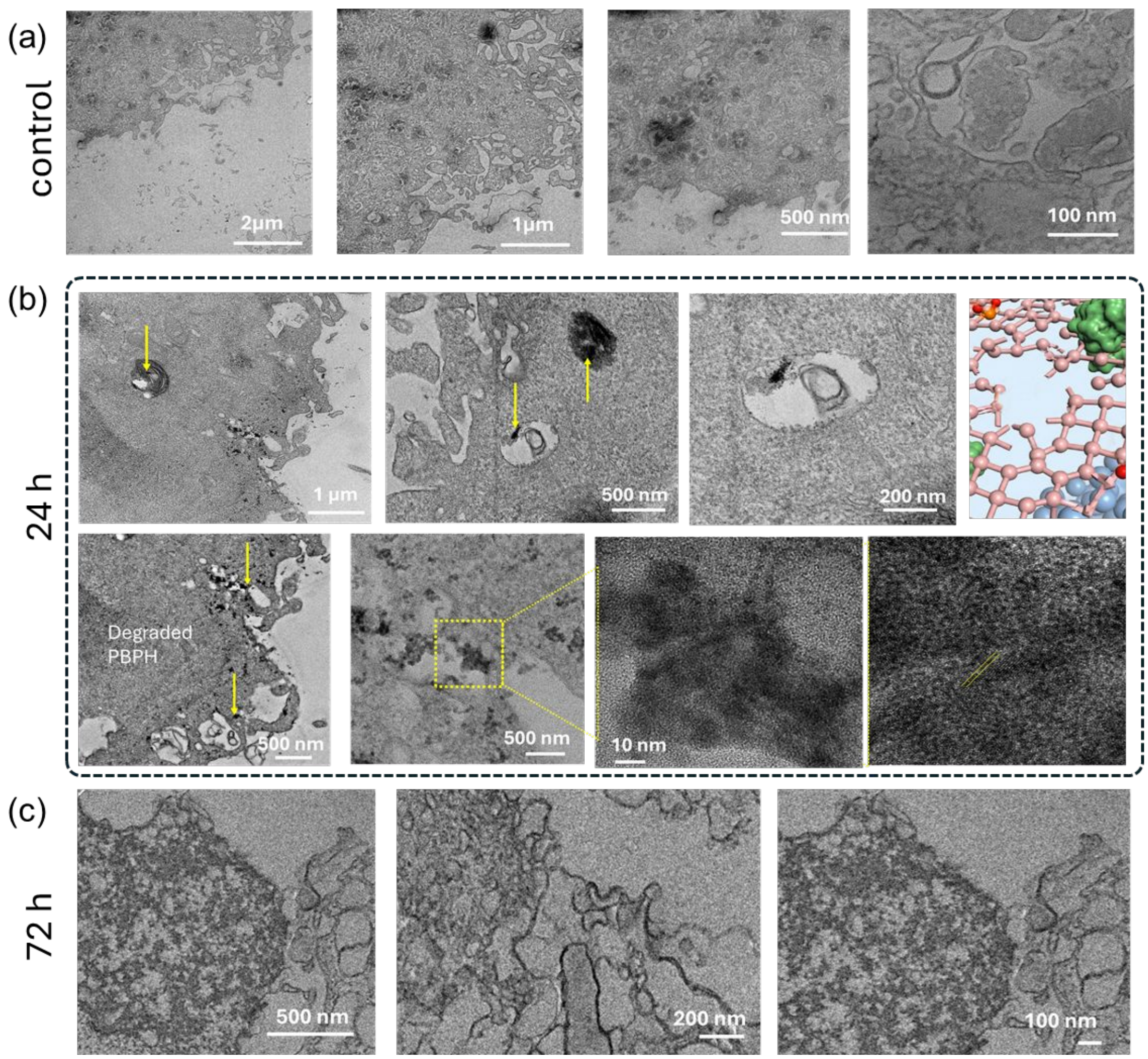

Figure S9. Time-resolved HRTEM analysis of intracellular PBPH exhibited (a) Control A549 cells showing no detectable borophene-associated electron-dense features, confirming absence of background signal. (b) PBPH-treated cells at 24 h displaying intracellular electron-dense regions (arrows) with partial structural disruption; inset schematic illustrates progressive ROS-mediated degradation of PBPH. (c) PBPH-treated cells at 72 h showing extensive loss of nanosheet morphology and absence of discernible lattice fringes, indicating intracellular degradation and conversion to amorphous boron-containing species.
